# Metabolic-related gene pairs signature analysis identifies ABCA1 expression levels on tumor-associated macrophages as a prognostic biomarker in primary IDH^WT^ glioblastoma

**DOI:** 10.3389/fimmu.2022.869061

**Published:** 2022-09-30

**Authors:** Shiqun Wang, Lu Li, Shuguang Zuo, Lingkai Kong, Jiwu Wei, Jie Dong

**Affiliations:** ^1^ Jiangsu Key Laboratory of Molecular Medicine, Medical School of Nanjing University, Nanjing, Jiangsu, China; ^2^ The Cancer Hospital of the University of Chinese Academy of Sciences (Zhejiang Cancer Hospital), Institute of Basic Medicine and Cancer (IBMC), Chinese Academy of Sciences, Hangzhou, Zhejiang, China; ^3^ Department of Nephrology, Affiliated Children’s Hospital of Zhejiang University, Hangzhou, Zhejiang, China; ^4^ Liuzhou Key Laboratory of Molecular Diagnosis, Guangxi Key Laboratory of Molecular Diagnosis and Application, Affiliated Liutie Central Hospital of Guangxi Medical University, Liuzhou, Guangxi, China

**Keywords:** primary glioblastoma, wild-type isocitrate dehydrogenase, metabolic-related gene pairs, prognosis, tumor-associated macrophages, ABCA1

## Abstract

**Background:**

Although isocitrate dehydrogenase (IDH) mutation serves as a prognostic signature for routine clinical management of glioma, nearly 90% of glioblastomas (GBM) patients have a wild-type IDH genotype (IDH^WT^) and lack reliable signatures to identify distinct entities.

**Methods:**

To develop a robust prognostic signature for IDH^WT^ GBM patients, we retrospectively analyzed 4 public datasets of 377 primary frozen tumor tissue transcriptome profiling and clinical follow-up data. Samples were divided into a training dataset (204 samples) and a validation (173 samples) dataset. A prognostic signature consisting of 21 metabolism-related gene pairs (MRGPs) was developed based on the relative ranking of single-sample gene expression levels. GSEA and immune subtype analyses were performed to reveal differences in biological processes between MRGP risk groups. The single-cell RNA-seq dataset was used to examine the expression distribution of each MRG constituting the signature in tumor tissue subsets. Finally, the association of MRGs with tumor progression was biologically validated in orthotopic GBM models.

**Results:**

The metabolic signature remained an independent prognostic factor (hazard ratio, 5.71 [3.542-9.218], *P* < 0.001) for stratifying patients into high- and low-risk levels in terms of overall survival across subgroups with MGMTp methylation statuses, expression subtypes, and chemo/ratio therapies. Immune-related biological processes were significantly different between MRGP risk groups. Compared with the low-risk group, the high-risk group was significantly enriched in humoral immune responses and phagocytosis processes, and had more monocyte infiltration and less activated DC, NK, and γδ T cell infiltration. scRNA-seq dataset analysis identified that the expression levels of 5 MRGs (ABCA1, HMOX1, MTHFD2, PIM1, and PTPRE) in TAMs increased with metabolic risk. With tumor progression, the expression level of ABCA1 in TAMs was positively correlated with the population of TAMs in tumor tissue. Downregulation of ABCA1 levels can promote TAM polarization towards an inflammatory phenotype and control tumor growth.

**Conclusions:**

The metabolic signature is expected to be used in the individualized management of primary IDH^WT^ GBM patients.

## Highlights

1. The metabolic signature can individually assess the prognosis of primary IDH^WT^ GBM patients.
2. Immune and metabolic processes were integrated into the molecular profiling descriptions of different GBM entities.

## Introduction

Since the WHO Classification of Central Nervous System tumors was revised in 2016, the diagnosis of glioma has developed into a new paradigm integrating molecular and histological features (1, 2). The mutation status of isocitrate dehydrogenase (IDH) is the primary biomarker for classifying distinct glioma entities. More than 90% of glioblastoma (GBM, WHO IV) patients have a wild-type IDH genotype (IDH^WT^), however, they currently lack robust prognostic biomarkers to further determine whether they benefit from chemoradiation (3). Therefore, the identification of prognostic factors in IDH^WT^ GBM patients is needed.

Despite ongoing efforts to define the prognostic molecular features of these patients (4–7), no biomarkers have been incorporated into routine clinical practice to date. Limitations are attributed to the lack of effective validation and overfitting of small discovery datasets; or the difficulty of multiple datasets merging to effectively eliminate batch effects from different techniques, laboratories, and samples (8). However, the elimination of batch effects is crucial for the robustness of the prognostic signature. Recently, a few studies have proposed new methods based on the relative ranking of gene expression levels to eliminate the biological variability of merging multiple datasets (9, 10).

Metabolic reprogramming is considered an emerging hallmark of cancer (11). Alterations in metabolism-related genes, such as IDH1 mutation, O^6^-methylguanine-DNA methyltransferase gene (MGMT) promoter methylation, or epidermal growth factor receptor (EGFR) amplification, are frequent in glioma patients and are closely related to prognosis (12–15). However, the prognostic performance of metabolic features in IDH^WT^ GBMs has not been adequately described. Therefore, extracting tumor hallmarks helps to outline the molecular features of patients and minimize data redundancy for pairwise ranking of full-size gene sets.

In this study, we integrated gene expression profiles of tumor tissue samples from multiple IDH^WT^ GBM datasets, and constructed and validated an individualized prognostic signature based on their metabolism-related genes.

## Materials and methods

### In silico study and public datasets

**Figure 1:**
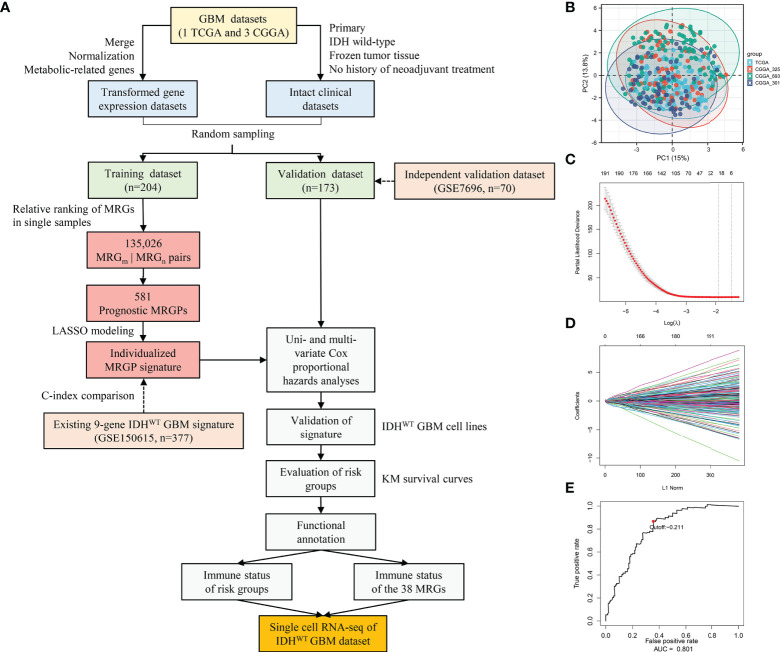
Construction and Evaluation of an Individualized Prognostic MRGPs Signature. **(A)** Overview of the study design. Four datasets were collected in the study, including one TCGA GBM dataset and three CGGA GBM datasets. The transformed gene expression matrix and clinical characteristics that removed nonconditional factors were integrated into a meta dataset and randomly divided into a training dataset (204 samples) and a validation dataset (173 samples). A total of 802 metabolism-related genes shared in the training and validation datasets were extracted for pairwise ranking in a single primary IDH^WT^ GBM sample. A total of 135,026 gene pairs were generated for each sample to construct an individualized MRGP prognostic model. Principal component analysis **(B)** was performed to evaluate batch effects of different pairwise transformed datasets. Each color represents a dataset, and every point comes from a sample. LASSO regression was performed to construct a prognostic model based on MRGPs. **(C)** 10,000-fold cross-validation for LASSO variable selection was plotted. Each red point indicates a λ value. The vertical line on the left represents the minimum error, and the vertical line on the right represents the maximum value of λ. **(D)** LASSO coefficients of prognostic MRGPs. **(E)** The 1-year time-dependent ROC curve for the MRGP signature in the training dataset. AUC represents the area under the curve.

In this study, we retrospectively analyzed the gene expression profiles of tumor tissue samples from four public glioblastoma (GBM) datasets, including one microarray dataset from the Chinese Glioma Genome Atlas (CGGA), two RNA-seq datasets from CGGA, and one RNA-seq dataset from The Cancer Genome Atlas (TCGA, **Table S1**). Only patients who met the following criteria were included: a) histologically confirmed grade IV glioma according to the WHO classification; b) fresh frozen tissue sample; c) no history of neoadjuvant therapy or other preoperative treatment; d) availability of isocitrate dehydrogenase (IDH) mutation status; and e) availability of data on overall survival, clinical annotation, genetics, and treatment information (16–18). Patients in CGGA_301 that overlapped with CGGA_325 and CGGA_693 (n = 24) were removed. Overall, a total of 377 patients were selected and divided into a training dataset (n = 204) and a validation dataset (n = 173). Further clinical characteristics of patients in each dataset are shown in **Table S2**. Details about the preprocessing of gene expression profiles and sample preparation used to obtain these datasets can be found in the **Supplemental Methods** or previous studies (19–21). The overall design of this study is shown in **Figure 1A**. The diagnostic accuracy study was based on the STARD guidelines and approved by the Institutional Review Committee of Nanjing University.

### Development of a prognostic signature based on single-sample MRGPs

To eliminate batch effects from different biological samples, we performed a pairing operation on 802 common metabolism-related genes (MRGs) for each sample. A new matrix of 135,026 MRG pairs (MRGPs) for 377 samples was generated. Details can be found in the **Supplemental Methods** or previous studies (9, 10). Principal component analysis (PCA) was further used to evaluate the platform or biological variability across different datasets.

In the training dataset, prognostic MRGPs were identified by using univariate Cox regression analysis to evaluate the association between each MRGP and patients’ overall survival (*P* < 0.001). A total of 581 prognostic MRGPs were selected to build a prognostic model by using the Lasso Cox proportional hazards regression model with 10-fold cross-validation (glmnet package, version: 3.0-2) (22). Robustness assessment of the metabolic signature against 1,000 randomizations of the training dataset can be found in the **Supplemental Methods**. A prognostic model including 21 MRGPs was constructed and used to calculate the risk score for each patient. Details of the 21 MRGPs can be obtained in **Table S4**. The formula of the risk score is as follows:


$$\text{Risk score}=\sum_{\text{i}=1}^{\text{n}}{\text{Coefficient}}_{\text{i}}\;\times {\text{ Value}}_{\text{i}}$$

Finally, we defined the optimum cut-off value for stratifying high- or low-risk groups by using a 1-year time-dependent receiver operating characteristic (ROC) curve (survival ROC package, version: 1.0.3).

### Evaluation and validation of the single-sample MRGPs signature

To determine whether the metabolic signature can be used as an independent prognostic factor in the management of IDH^WT^ GBM, we performed uni- and multivariate Cox proportional hazards analyses on patients in the training and validation datasets (**Table S5**). Age, gender, MGMTp methylation status, and risk were coded as continuous variables (e.g., female was coded as 0, male as coded as 1; MGMTp methylation was coded as 0, MGMTp unmethylation was coded as 1).

Furthermore, we evaluated the prognostic accuracy of our MRGPs signature and one existing 9-gene IDH^WT^ GBM signature in a continuous form by using the concordance index (C-index). Details about the C-index comparison can be found in the **Supplemental Methods**. Kaplan–Meier curve analysis was used to validate the overall survival stratification of our MRGPs signature and 9-gene signature in the training and validation datasets.

In the training and validation datasets, we utilized a Pearson correlation heatmap (pheatmap, version: 1.0.12) to explore the expression correlation of 38 MRGs that make up the prognostic signature. Considering that the main population of tumor tissues was tumor cells, we performed real-time PCR to identify the expression profile of 38 MRGs of two human IDH^WT^ GBM cell lines (U87-MG and U251-MG) and three human control cell lines (HeLa S3, HEK293, and HA1800) and to mimic risk decisions based on the metabolic risk score. Details about cell lines, RNA isolation, PCR, and identification of IDH1 mutations can be found in the **Supplemental Methods**.

### Functional annotation and enrichment analyses

To reveal the biological significance of the MRGPs signature, we conducted Gene Ontology (GO) functional annotation analysis of its component MRGs with the Database for Annotation, Visualization and Integrated Discovery (DAVID) Bioinformatics Resources database (https://david.ncifcrf.gov/). Significant GO biological processes (*P* < 0.05) were detected (**Table S6**). In addition, the meta dataset was divided into high- and low-risk groups according to MRGPs, and gene set enrichment analysis (GSEA) was performed on these groups (fgsea package, version: 1.12.0; C5.bp.v7.1; 10,000 permutations). Significantly enriched biological processes (*P* value < 0.05) were examined.

### Tumor purity and immune infiltration analyses

Tumor purity possesses important clinical implications in glioma classification (23). Estimation of STromal and Immune cells in MAlignant Tumor tissues using Expression data (ESTIMATE) analysis was performed to estimate differences in tumor purity between MRGP risk groups in the meta dataset (estimate package, version: 1.0.13). The ESTIMATE score was considered to be negatively correlated with tumor purity. Pearson correlation analysis was used to determine the correlation between tumor purity and the expression levels of 38 MRGs in risk groups in the meta dataset.

We further explored the immune infiltration status of the metabolic high- and low-risk groups by using xCell (https://xcell.ucsf.edu/) and Cell-type Identification by Estimating Relative Subsets of RNA Transcripts (CIBERSORT package, version: 1.03). Specifically, the normalized gene expression matrix in the meta dataset was divided into high- and low-risk groups based on the MRGPs signature. The relative abundances of immune cells between MRGP risk groups were identified (matrix at 1,000 permutations). The profile of immune infiltration of different risk groups was displayed by the radar chart (fmsb package, version: 0.7.1). Pearson correlation analysis was used to determine the correlation between the relative abundances of immune cells and the expression levels of 38 MRGs in risk groups in the meta dataset.

### Expression distribution of the 38 MRGs in the single-cell RNA-seq dataset

A single-cell RNA-seq dataset for IDH^WT^ GBM (GEO: GSE131928) was downloaded from the Single Cell Portal (https://singlecell.broadinstitute.org/). Data processing as previously described (24). Briefly, use with arguments “-q –phred33-quals -n 1 -e 99999999 -l 25 -I 1 -X 2000 -a -m 15 -S -p 6”. Expression values were calculated by RSEM v1.2.3 in paired-end mode, using the parameter “–estimate-rspd –paired end -sam -p 6”, from which TPM values for each gene were extracted. For cells annotations treated with 10X, we used CellRanger with default parameters. The dataset included 24131 cell sequencing data points from 28 tumor samples (24). Scaled mean expression data (robust z score) were used to identify the expression levels of 38 MRGs in 28 samples. Furthermore, the risk level of each single-cell sequencing sample was defined by our MRGP model (**Figure S8**). Four main cell populations were found in all single cells, including macrophages, malignancies, oligodendrocytes, and T cells. t-distributed stochastic neighbor embedding (tSNE) was performed to plot the expression distribution of all 38 MRGs in the four populations. A heatmap was generated to show changes in the expression levels of 38 MRGs from low risk to high risk in the four populations.

### MRGPs risk coefficient connection and protein–protein interaction network

The Search Tool for the Retrival of Interacting Genes/Proteins (STRING, https://www.string-db.org/) database was used to identify the direct interaction network between 38 proteins. To reveal the inherent association of the metabolic signature, we used Cytoscape (version 3.7.2) to draw a network diagram for these MRGs.

### Cell lines

The human glioblastoma (GBM) cell lines (U87-MG and U251-MG), the human cervical carcinoma cell line (HeLa S3), the human embryonic kidney cells 293 (HEK293), and human normal astrocyte cell line (HA1800) were purchased from ATCC, tested for mycoplasma contamination, and authenticated by short tandem repeat (STR) analysis. The murine GBM cell line GL261 was purchased from the China Center for Type Culture Collection. TAMs were isolated by anti-mouse F4/80 MicroBeads UltraPure (Miltenyi Biotec, 130-110-443) from GL261 tumors. Bone marrow-derived macrophages (BMDMs) were obtained by *in vitro* M-CSF differentiation of C57 mouse bone marrow cells. All GBM cells were cultured in DMEM/F12 medium (D8437, Sigma); HeLa S3, HEK293, and HA1800 cells were cultured in DMEM containing 4.5 g/L glucose (Cat No. 11965-092, Gibco) supplemented with 10% fetal bovine serum (Cat No. 10099-141, Gibco), 100 U/mL penicillin, and 100 μg/mL streptomycin (Cat No. 15140-122, Gibco). All cells were maintained at 37°C in a humidified incubator with 5% CO2.

### Animal studies

Animal care and handling procedures were carried out following the NIH Guide for the Care and Use of Laboratory Animals and were approved by the Institutional Review Board of Nanjing University. For the intracranial GBM model, 6- to 8-week-old male C57BL/6 mice and NOD-Prkdcscid Il2rgnull (NCG) mice were purchased from Nanjing University Model Animal Institute. As described in a previous study, the mice were anesthetized, and dissociated GBM cells were implanted into 2.0 mm depth to the skull 1.0 mm anterior and 2.0 mm lateral to bregma by using a stereotactic apparatus (25). GL261^IDH-WT^ cells (2 x 10^5^ cells/2 μl PBS) were intracranially inoculated into the caudate nucleus of C57BL/6 mice. U87-MG^IDH-WT^ cells (5 x 10^5^ cells/4 μl PBS) were injected into NCG mice. The survival and neurological symptoms of the mice were monitored every other day. To assess the mRNA expression levels of the 5 MRGs of TAMs during tumor progression, orthotopic GL261^IDH-WT^-bearing mice were sacrificed on days 7, 14, and 21, and tumor tissues were collected for magnetic bead sorting of TAMs. The isolated TAMs were further extracted for total RNA and the expression of these genes was performed by qPCR. To assess the expression levels of ABCA1 on TAMs during tumor progression, orthotopic GL261^IDH-WT^- or U87-MG^IDH-WT^-bearing mice were sacrificed on days 7, 14, and 21, and tumor tissues were collected for FCM analysis. To assess the association between cholesterol metabolism and ABCA1 expression levels in macrophages, differentiated BMDMs were ex vivo treated with vehicle, 1 ug/ml and 10 ug/ml cholesterol (Sigma, C4951) for 24 hours for FCM analysis; orthotopic GL261^IDH-WT^-bearing mice were sacrificed on days 17 to isolate TAMs, and these sorted TAMs were ex vivo treated with vehicle (DMSO), 2 uM and 5 uM lovastatin (Sigma, 75330-75-5) for 24 hours for FCM analysis. To assess the therapeutic activity of modulating ABCA1 expression, GL261^IDH-WT-Luc^ cells (2 x 10^5^ cells/2 μl PBS) were intracranially inoculated into the caudate nucleus of C57BL/6 mice. On day 7 after tumor inoculation, mice were given oral gavage with vehicle (0.5% methylcellulose, 2% Tween-80 in water) or 10 mg/kg lovastatin (NJDULY, A0157) daily for 14 days. At the end of dosing, tumor growth was examined by an *In Vivo* Imaging System (IVIS, LB 983 NC100). To assess TAMs polarization following modulation of ABCA1 expression, FCM analysis of TAMs inflammatory factor expression was performed in lovastatin-treated GL261^IDH-WT-Luc^ tumors for 14 days as previously described.

### Flow cytometry

For *in vivo* macrophage analysis, tumor tissue was collected at set time points after tumor cell engraftment, digested, and filtered through a 70 μm strainer. Dissociated cells were further incubated with the following antibodies: anti-mouse CD16/CD32 (Multi Sciences, clone 2.4G2, Cat No. AM016-100), IgG2a, κ isotype ctrl (Biolegend, clone MOPC-173, Cat No. 400233), anti-mouse ABCA1 (BIO-RAD, clone 5A1-1422, Cat No. MCA2681), anti-mouse F4/80 (Biolegend, clone BM8, Cat No. 123110), anti-mouse\human CD11b CM1/70, Cat No. 101229), anti-mouse CD86 (Biolegend, clone GL-1, Cat No. 105005), anti-mouse CD206 (Biolegend, clone C068C2, Cat No. 141715), anti-mouse TNF-α (Biolegend, clone MP6-XT22, Cat No. 506303), anti-mouse IFN-γ (Biolegend, clone XMG1.2, Cat No. 506303), anti-mouse Arginase 1 (Abbexa, Polyclonal, Cat No. abx319179), and anti-mouse CD45 (Biolegend, clone 30-F11, Cat No. 103112). Intracellular staining was done using Fixation/Permeabilization kit (BD, 554722). Samples were subjected to FCM by using BD FACS Calibur, BD Aria I, and Beackman CytoFLEx. Data were analyzed with FlowJo (vX.0.7).

### Statistical analysis

Statistical analyses were performed by using R software (version: 3.6.3; https://www.r-project.org/) or GraphPad Prism (v. 8.0.1). Continuous variables were compared by using Student’s t test, the Mann–Whitney test, or the Wilcoxon rank-sum test. Cumulative survival analyses were performed using the Kaplan–Meier method, and the survival differences were analyzed using the log-rank test (survival package, version: 3.1-12). In the univariate and multivariate analyses, the Wald test was used to assess the association of the MRGP and clinicopathologic factors with overall survival. Pearson correlation tests were performed to assess the correlation of MRGs with tumor purity or immune cell abundances (corrplot package, version: 0.84).

## Results

### Development and definition of MRGPs signature based on single IDH^WT^ GBM samples

In this retrospective study, a total of 377 IDH^WT^ GBM patients (236 female, 141 male) were selected from the TCGA and CGGA databases according to the criteria shown in **Figure 1A**. Patients were further randomly assigned to the training dataset (n = 204) and validation dataset (n = 173). No significant differences in clinical characteristics between the training and validation datasets were observed (**Table S2**). Among the 3,679 MRGs we obtained in the KEGG database, a total of 802 MRGs shared with all datasets were identified, and 135,026 MRGPs were further constructed for each sample. Details about the elimination of platform bias and biological variability can be found in the **Supplementary Methods**. The PCA plot (**Figure 1B**) indicated that four datasets had no significant clustering after normalization and pairing.

In the training dataset, we evaluated and obtained 581 MRGPs related to overall survival (OS). Then, on the basis of these prognostic MRGPs, we performed Lasso Cox proportional hazard regression to construct a prognostic signature consisting of 21 MRGPs (**Figures 1C, D**). The 21 MRGPs signature was composed of 38 unique MRGs. The coefficient values of 13 MRGPs out of 21 MRGPs (62%) were > 0.05 or < -0.05, indicating higher prognostic power (**Table S4**). We further assessed the robustness of the MRGPs signature, and its frequency was significantly higher than the frequency obtained by 1000 randomizations (*P* < 0.001, **Supplemental Results**). The optimum cut-off value for MRGPs risk stratification was identified to be -0.211 by using a 1-year time-dependent ROC curve analysis (area under the curve [AUC] = 0.801; **Figure 1E**).

### Validation of the MRGPs signature as an independent prognostic factor

**Figure 2:**
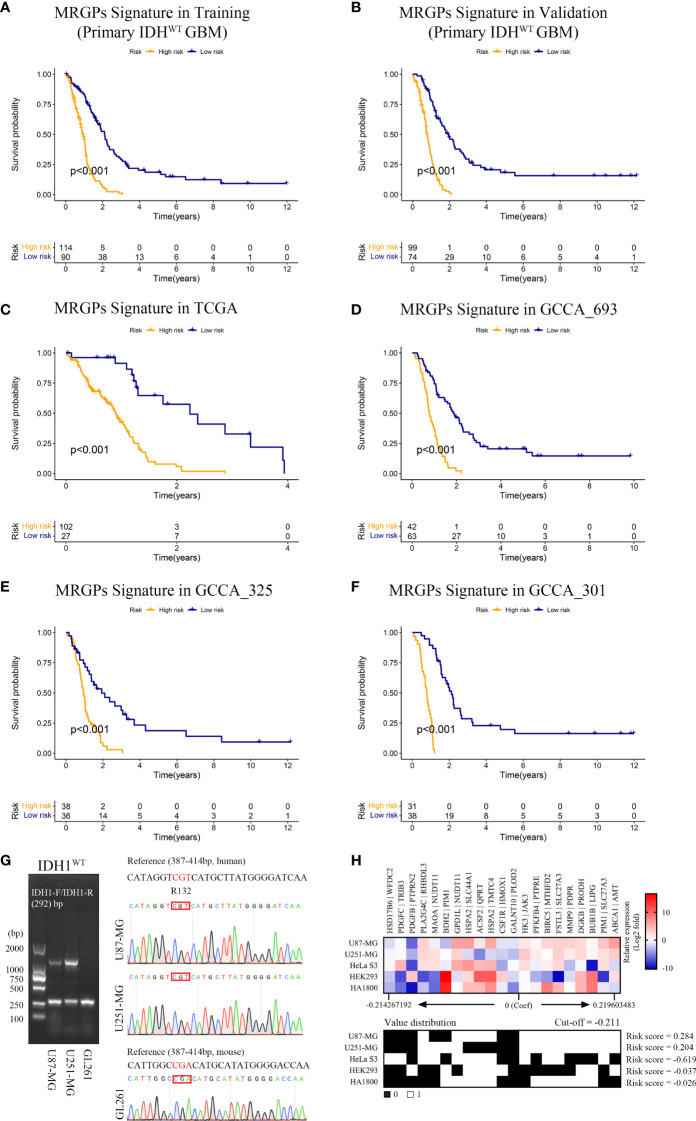
MRGPs Signature Stratifies the Overall Survival of IDH^WT^ GBM With Different MRGP Risks. Kaplan–Meier curves of overall survival in IDH^WT^ GBM patients in the MRGP risk groups. The overall survival of patients in the training **(A)** and validation **(B)** datasets was stratified by the MRGP risk score. The overall survival of IDH^WT^ GBM in TCGA **(C)**, CGGA_693 **(D)**, CGGA_325 **(E)**, and CGGA_301 **(F)** datasets was stratified into high- and low-risk groups based on MRGP risk score (*P* values are all < 0.001, log-rank test). **(G)** Identification of IDH1 gene status in human GBM cells. No mutations were observed at the R132 site in the U87-MG and U251-MG cell lines. **(H)** Validation of the predictive efficacy of the MRGPI for human GBM cell lines. RT–PCR assays were performed to identify the expression levels of 38 MRGs relative to GAPDH in GBM cell lines and the control cell lines (HeLa S3, HEK293, and HA1800). The relative expression data between MRGs were used to calculate the risk level of cell lines *in vitro*. The cut-off value is equal to -0.211. GBM cell risk scores were all greater than the cut-off value (U87-MG = 0.284, U251-MG = 0.204), indicating high risk.

Next, we conducted a comprehensive evaluation of the prognostic power of the MRGPs signature. The MRGP signature significantly stratified patients into high- and low-risk groups in terms of OS in the training and validation datasets (**Figures 2A, B**), their four original datasets [TCGA (**Figure 2C**), CGGA_693 (**Figure 2D**), CGGA_325 (**Figure 2E**), and CGGA_301 (**Figure 2F**)], and an external independent validation dataset [GSE7696 (**Figure S1**)]. We also found that the survival of high-risk patients with upper quartile risk scores was worse than that of low-risk patients with lower quartiles in the training dataset (*P* < 0.001, **Figure S2**). Univariate Cox proportional hazards analysis demonstrated that the metabolic signature was a high-risk factor in the prognosis of patients (hazard ratio [HR] ranged from 5.921 [95% CI, 3.703-9.470; *P* < 0.001] to 6.676 [95% CI, 4.526-9.849; *P* < 0.001]; **Table S5**). After adjusting for clinical factors such as age, gender, and MGMTp methylation status, we further determined that the metabolic signature was an independent prognostic factor in multivariate analysis. The HR ranged from 5.714 [95% CI, 3.542-9.218; *P* < 0.001] to 6.698 [95% CI, 4.478-10.018; *P* < 0.001]; **Table S5**). Here, although the covariate of MGMTp methylation status in multivariate analysis was not statistically significant (*P* = 0.533 [training]; *P* = 0.681 [validation]), the prognostic accuracy of the MRGPs signature in the validation dataset was improved for MGMTp nonmethylated patients (C-index = 0.801 [0.751] - [0.852]) compared with MGMTp methylated patients (C-index = 0.648 [0.564] - [0.733]; **Figure S3**). Furthermore, we also evaluated the survival stratification efficacy of the MRGPs signature in classical, mesenchymal, neural, and proneural GBM subtypes in the training and validation datasets (**Figure S4**). In addition to the neurotype subtype (*P* = 0.041 [training]; *P* = 0.865 [validation]; C-index range from 0.500 to 0.650), our signature achieved significant survival stratification efficiency and accuracy for the other three subtypes (*P* ≤ 0.032 in training and validation datasets; C-index range from 0.699 to 0.793).

Furthermore, we compared our MRGP signature with one recently developed 9-gene IDH^WT^ GBM biomarker in continuous form, the C-index, in the training and validation datasets (**Figure S5**). Our signature achieved superior performance to that of the 9-gene signature on both the training and validation datasets (mean C-index 0.73 vs. 0.57).

Given that malignant tumor cells account for the main population of the sequenced tumor tissue samples and the high expression correlation between 38 unique MRGs composed of the MRGPs signature (**Figure S6**), we tried to mimic and validate the reliability and specificity of the prognostic model at the *in vitro* cell line level (**Figures 2G, H**). Compared with the control cell lines (HeLa S3, HEK293, and HA1800), our signature showed consistent risk prediction results in two IDH^WT^ GBM cell lines (risk score = 0.284 [U87-MG]; risk score = 0.204 [U251-MG]).

**Figure 3:**
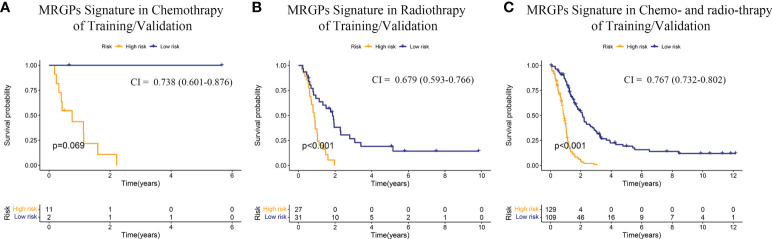
Kaplan–Meier Overall Survival Curve Analysis of the Responses of IDH^WT^ GBM With Different MRGP Risks to Chemotherapy/Radiotherapy. Patients from the training and validation datasets were integrated into a meta-dataset. Patients receiving chemotherapy only **(A)**, radiotherapy only **(B)**, and radiotherapy combined with chemotherapy **(C)** were further divided into high- and low-risk groups based on the MRGP score. Kaplan–Meier overall survival curves were generated to show the risk stratification of the prognostic model. CI indicates the C-index, which was used to evaluate the accuracy of the prognostic model in datasets. The performance of the MRGP model in the combination therapy group (CI = 0.767, *P* < 0.001) was superior to that in the single therapy groups (Chem, CI = 0.738, *P* = 0.069; Radio, CI = 0.679, *P* < 0.001).

Finally, we evaluated the survival stratification efficacy of the signature on patients with postoperative chemotherapy (temozolomide) and/or radiotherapy regimens in the integrated meta dataset (**Figure 3**). The prognostic accuracy of the MRGPs signature for patients with radiotherapy and chemotherapy was superior to that of single treatment (C-index: 0.767 [chem + radio] vs. 0.738 [chem only] or 0.679 [radio only]). In summary, our MRGP signature could soundly predict the prognosis of patients with IDH^WT^ GBM.

### Functional annotation of the MRGPs signature

**Figure 4:**
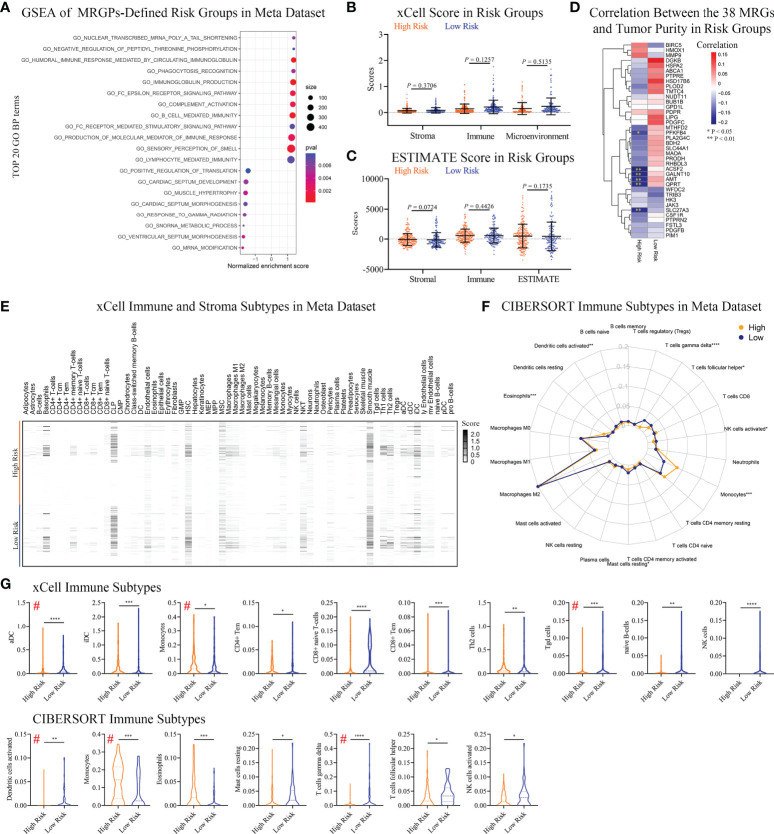
Differences in Immune Infiltration Profiles Between Risk Groups Defined by Metabolic Signature. **(A)** GSEA of MRGP risk groups in the meta dataset (*P* < 0.05). The top 20 GO biological processes are shown. Multiple GO biological processes related to immunology, including immunoglobulin-mediated responses, were enriched in high-risk patients. **(B, C)** xCELL **(B)** and ESTIMATE **(C)** analyses of tumor purity between MRGP risk groups in the meta dataset. No significant differences between risk groups were observed (Mann–Whitney test). **(D)** Pearson correlation heatmaps of the expression of 38 MRGs and tumor purity in the risk groups of the meta-dataset. Value > 0 indicates that gene expression is positively correlated with tumor purity, indicating less immune infiltration. *P < 0.05; **P < 0.01. **(E)** xCell heatmap of the abundance of 64 immune and stroma cells in IDH^WT^ GBM patients within risk groups in the meta dataset. **(F)** CIBERSORT analysis of the abundance of 22 immune cells in IDH^WT^ GBM patients within risk groups in the meta dataset. **(G)** Immune cell subtypes significantly different between risk groups. Data is related to **(E, F)**. # indicates the same immune subtype in xCell and CIBERSORT analyses. The difference between risk groups was calculated by the Mann–Whitney test (*P < 0.05, **P < 0.01, ***P < 0.001, ****P < 0.0001).

We conducted a GO annotation analysis on the 38 MRGs composed of the signature in DAVID (**Table S6**). Most biological processes were focused on phosphorylation (red underline) and lipid metabolic processes (blue underline). Interestingly, immune-related process macrophage differentiation was also enriched (green underline). Thus, we further performed GSEA on MRGPs risk groups in a meta dataset to explore potential biological differences (**Figure 4A**). We found that the top 20 GO biological process terms (*P* < 0.05) included not only phosphorylation but also various immune-related processes, such as humoral immune response and phagocytosis processes, which was enriched in the high-risk group.

### Differentiation of immune infiltrating subgroups between different risk groups based on the MRGPs signature

Given that our metabolic signature was closely related to multiple immune processes, we tried to further discover the association between risk groups or the corresponding 38 MRGs and patient immune status. No significant difference in tumor purity between risk groups was observed (**Figures 4B, C**). There was only a low negative correlation between the expression levels of the 6 MRGs and tumor purity in high-risk patients (**Figure 4D**). We found a significant difference in the percentage of necrosis between the risk groups in the bottom sections of the TCGA dataset (**Figure S7**). The average level of necrosis in the high-risk group was ~2-fold that of the low-risk group (9.9% vs. 5.5%, *P* < 0.05), suggesting a change in immune status.

To reveal immune status between risk groups defined by MRGPs, we performed xCELL and CIBERSORT subtypes analyses. The results indicated that the MRGPs signature can profile the abundance of immune cell infiltration in patients with different risks (**Figures 4E, F**) and showed that MRGP-defined high-risk patients had more monocytes and less activated DC and T cell gamma delta (γδ T cell) infiltration in the TME (**Figure 4G**). For example, in CIBERSORT analysis (**Figure 4G** lower panel), high-risk patients had higher monocyte abundance than low-risk patients (14.4% vs. 7.5% immune cells, *P* < 0.001). Studies have shown that the cell density of glioma-infiltrating microglia/macrophages (GAMs) is related to the degree of malignancy of gliomas and gradually increases with progression (26, 27). Interestingly, our study indicated that although the macrophage family dominated the population of infiltrating immune cells, there was no difference in abundance between the two groups (**Figures 4E–G**).

To reveal the association of MRGPs signature with immune subtypes, we performed Pearson correlation analysis on 38 MRGs and 22 immune cells (**Figure S8**). The heatmap indicated that the expression level of MRGs had a significant correlation with the abundance of immune cells. We found that the MRGPs signature can also characterize the molecular profile of immune cells with no significant differences in abundance in different risk groups. For example, in low-risk patients, M2 macrophage (31.4% vs. 31.7%) abundance was positively correlated with the expression levels of PLA2G4C, GALNT10, BDH2, AMT, SLC44A1, HSPA2, and FSTL3 (coefficient > 0.3), while in the high-risk group, there was no obvious correlation.

### Expression distribution of the 38 MRGs in the single-cell RNA-seq dataset

**Figure 5:**
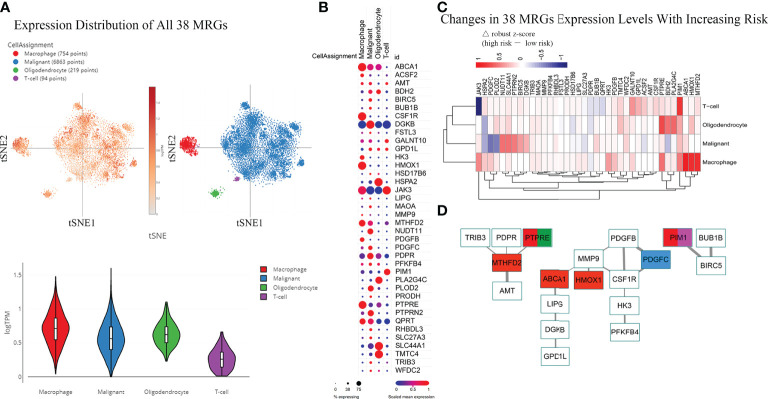
Differences in the Expression Distribution of 38 MRGs Between Risk Groups in the Single-cell RNA-seq IDH^WT^ GBM Dataset. The risk level of each sample in the single-cell RNA-seq IDH^WT^ GBM dataset (n = 28) was defined based on the relative expression distribution of the 38 MRGs (**Figure S8**). The data indicated that four main cell types are present in GBM tissue: macrophages, malignancies, oligodendrocytes, and T cells. **(A)** Overall expression distribution of signature genes (all 38 MRGs) in four main cell types. **(B)** Expression distribution of 38 MRGs in four main cell types. **(C)** Expression alterations of 38 MRGs in four main cell types with increased metabolic risk. **(D)** Protein–protein interaction network of the 38 MRGs. The thickness of the line indicates the level of the combined score (0.4~1.0). Red indicates MRGs that are differentially expressed in macrophages between different risk groups (|△ robust z score| > 0.5, **(C)**. Blue indicates the MRG in malignant cells. Green indicates the MRG in oligodendrocytes. Purple indicates the MRG in T cells.

Above, we determined the association between infiltrating immune cells and the molecular profile of the MRGPs signature in different risk groups. However, their expression distribution in tumor tissues is not yet clear. Thus, we tried to retrieve the expression distribution of 38 MRGs in the IDH^WT^ GBM single-cell sequencing dataset and to calculate the risk score for each sample according to the risk cut-off (**Figure S9**). Twenty samples (71.4%) were defined as high risk. Four main cell populations in tumor bulks were identified, including macrophages, malignant cells, oligodendrocytes, and T cells. The main population of tumor-infiltrating immune cells shown in scRNA-seq data was consistent with our immune subtype analysis, in which the main populations of immune infiltrating cells in patients with IDH^WT^ GBM were monocytes/macrophages and T-cell families (**Figure 4F**). More expression distribution for all 38 MRGs was identified in macrophages, malignant cells, and oligodendrocytes and less in T cells (**Figure 5A**). The distribution of each MRG is shown in **Figure 5B**. Furthermore, according to the risk grouping, the expression changes of 38 MRGs were identified (**Figure 5C**). In macrophages, 5 MRGs (ABCA1, HMOX1, MTHFD2, PIM1, and PTPRE) were identified as having significant expression differences between the risk groups. The 4 MRGs (NUDT11, PDGFC, PLOD2, and SLC44A1) in malignant cells. The 3 MRGs (BDH2, PLA2G4C, and PTPRE) in oligodendrocytes. The 2 MRGs (JAK3 and PIM1) in T cells.

The PPI network of the 38 MRGs demonstrated that 19 MRGs constituted 3 potential interactive modules (**Figure 5D**). The 19 MRGs included 5 differentially expressed MRGs from macrophages (red color), 1 MRG from malignant cells (blue color), 1 MRG from oligodendrocytes (green color), and 1 MRGs from T cells (purple color). The in-silico study suggests that MRGPs-based survival risk stratification is closely related to changes in the abundance and/or molecular features of the monocyte/macrophage family.

### Biological validation of the correlation between tumor-associated macrophage ABCA1 expression and IDH^WT^ GBM progression

**Figure 6:**
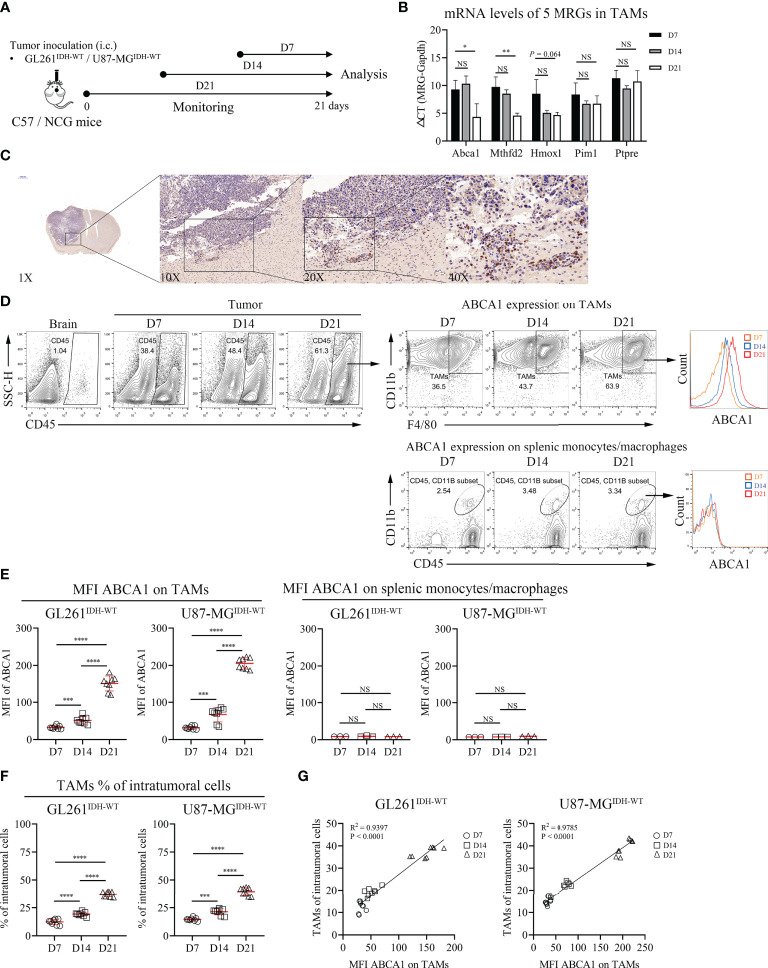
The Expression Level of ABCA1 in TAMs Positively Correlated with the Population of TAMs with IDH^WT^ GBM Tumor Progression. **(A)** Experimental setup to study the links between MRGs expression in macrophage and tumor progression. **(B)** Quantitative analysis of mRNA expression levels of 5 MRGs in TAMs with tumor progression. C57 mice were intracranially inoculated with GL261 cells. On day 7, day 14 and day 21, mice were sacrificed and collected tumor tissues. TAMs were isolated from tumors by anti-F4/80 microbeads and subjected to qPCR to detect mRNA expression (n = 3). **(C)** Immunohistochemical analysis of the expression levels of ABCA1 in mouse tumor and normal brain tissue. **(D, E)** Representative flow cytometry plots **(D)** and quantitative analysis **(E)** of TAMs and peripherally splenic monocytes/macrophage ABCA1 expression levels with tumor progression. Orthotopic tumors were collected on days 7, 14 and 21 after tumor inoculation. n = 8. f-g: Quantitative analysis of TAM populations **(F)** and pearson correlation analysis of TAM populations and TAM ABCA1 expression levels **(G)**. Data is related to **(D, E)**. The difference between risk groups was calculated by the Mann–Whitney test (NS, no statistical significance, *P < 0.05, **P < 0.01, ***P < 0.001, ****P < 0.0001).

There is abundant clinical and experimental evidence that strongly links increased numbers of TAMs with poor prognosis (28). However, metabolism in TAMs and GBM tumorigenesis or prognosis remain poorly understood. Thus, we performed further biological validation to determine whether the 5 MRGs identified in TAMs were associated with the development of IDH^WT^ GBMs (**Figure 6A**). We found that the mRNA expression levels of ABCA1 and MTHFD2 genes in TAMs increased significantly with tumor progression (**Figure 6B**). Given that ABCA1 is among the gene pairs with the highest risk factor compared to MTHFD2 (**Table S4**; MRGP-01 = 0.2196 vs. MRGP-07 = 0.0248), we further explored the potential biological associations between ABCA1 and TAMs. In IHC of brain tissue from GL261 tumor-bearing mice, we identified that the expression level of ABCA1 in tumor tissue was higher than that in normal brain tissue (**Figure 6C**). Furthermore, in the distribution analysis of ABCA1 expression on tumor cells and tumor-infiltrating immune cells, we found that the expression level of ABCA1 in TAMs was significantly higher than that in TILs and monocytes (**Figure S10**). And in two IDH^WT^ GBM models, we found that both the expression level of ABCA1 on TAMs and the intratumoral population of TAMs increased with tumors progression (**Figures 6D–F**) and showed a high positive correlation between them (**Figure 6G**). These results demonstrate that the expression level of ABCA1 on TAMs was a risk factor for IDH^WT^ GBMs.

**Figure 7:**
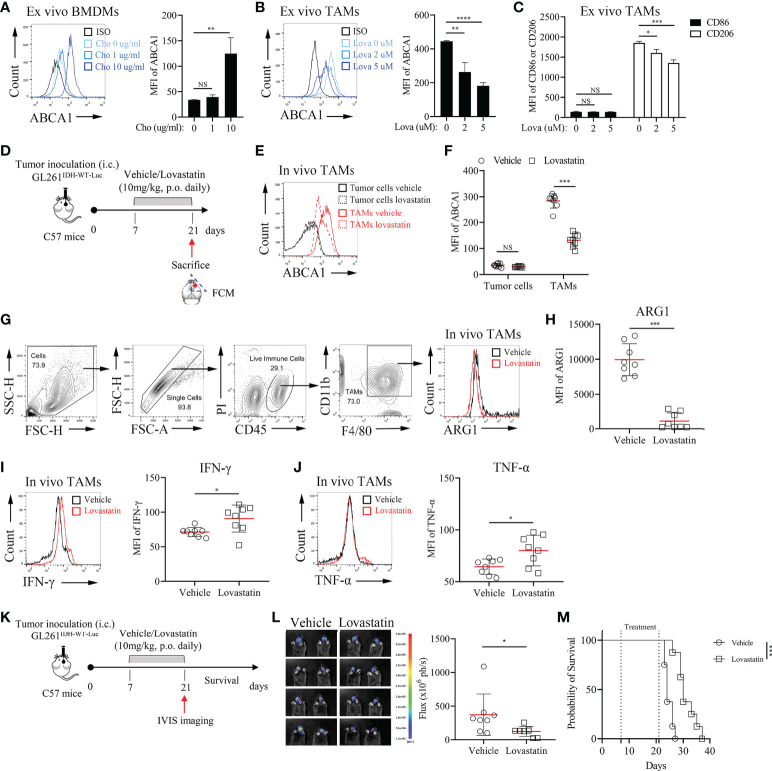
Pharmacological Inhibition of ABCA1 Enhances the Inflammatory Polarization of TAMs *In Vivo***(A)**: Quantification of ABCA1 expression in cholesterol-treated BMDMs ex vivo. BMDMs were treated with the indicated concentrations of cholesterol for 24 h. Cells were harvested and FCM was performed to identify the expression level of ABCA1 (n = 3). **(B, C)**: Quantification of ABCA1 expression **(B)** and CD86 and CD206 expression **(C)** in lovastatin-treated TAMs ex vivo. TAMs were treated with the indicated concentrations of lovastatin for 24 h. FCM was performed to identify these molecular expressions. **(D)**: Experimental setup of lovastatin-treated murine IDH^WT^ GBM model. Eight mice per group. **(E, F)**: Representative flow cytometry plots **(E)** and quantification of ABCA1 expression levels in TAMs **(F)**. **(G–J)**: Representative flow cytometry plots **(G)** and quantification of TAM functional polarization **(H–J)**. ARG1 **(H)** were identified as anti-inflammatory macrophage markers; IFN-γ **(I)** and TNF-α **(J)** were identified as inflammatory macrophage markers. **(K–M)**: Experimental setup **(K)** and tumor growth and survival monitoring **(L, M)** of lovastatin-treated murine GL261^IDH-WT-Luc^ model. Eight mice per group. The difference between risk groups was calculated by the Mann–Whitney test and log-rank test (NS, no statistical significance, *P < 0.05, **P < 0.01, ***P < 0.001, ****P < 0.0001).

Annotation analysis of MRGs indicated that ABCA1 was related to lipid metabolism- related processes (**Table S6**). ABCA1 uses cholesterol as its substrate to mediate cholesterol efflux in the cellular lipid removal pathway (29). Thus, we further investigated whether modulating cellular cholesterol levels significantly alters ABCA1 expression in macrophages and whether reducing ABCA1 expression levels in TAMs affects tumor progression. *In vitro* cholesterol loading assay showed that modulating cholesterol levels in macrophages can significantly affect their ABCA1 levels (**Figure 7A**). Lovastatin treatment *in vitro* and *in vivo* reduced ABCA1 levels in TAMs (**Figures 7B, D–F**) and shifted TAMs functional specialization from an inhibitory to a pro-inflammatory phenotype (**Figures 7C, G–J**). Finally, to determine whether ABCA1 can be an effective target for the treatment of IDH^WT^ GBMs, we performed a lovastatin treatment experiment in the orthotopic GL261^IDH1-WT-Luc^ model and showed that this modulation of immune metabolism can significantly control tumor progression (**Figures 7K–M**). These results confirm the reliability of our constructed metabolic signature and indicate a crucial role for macrophage lipid-related metabolism in maintaining malignant progression.

## Discussion

Patients with IDH^WT^ GBM are at high risk of recurrence, even with postoperative chemoradiotherapy. Intratumoral heterogeneity allows patients to respond differently to the same intervention (24, 30). Therefore, reliable prognostic biomarkers are urgently needed to identify patients who may benefit from additional therapy and who may be at risk of recurrence. Significant research on prognostic molecular signatures has led to breakthroughs in the estimation of survival in GBM patients (4, 31–34), but their accuracy in the IDH^WT^ subgroup remains limited. In this study, we developed an individualized prognostic signature of primary IDH^WT^ GBM based on 21 MRGPs, and validated its prognostic value in multiple independent datasets. Our metabolic signature can further stratify patients into distinct survival risk subgroups, when considering other clinical variables (e.g., clinical care, MGMTp methylation status, and expression subtypes). In the combination index comparison, our metabolic signature exhibited superior accuracy compared with another 9-gene IDH^WT^ GBM signature (31).

To identify reliable prognostic biomarkers for IDH^WT^ GBMs, we integrated gene expression profiles from four datasets and employed a relative ranking method that is based on gene expression levels and specifically designed to robustly eliminate technical and sampling biases (9, 35). As such, our metabolic signature can individually assess the prognosis of IDH^WT^ GBM and may be easily translated into the clinic.

Discovery of novel biomarkers associated with metabolic reprogramming in GBM tumors might have important implications for identifying potential molecular targets and developing precision medicine (36–38). He et al. discovered that glycolysis, gluconeogenesis and oxidative phosphorylation processes differ significantly among GBM patients with different prognoses (15). Patients with wild-type or mutant IDH GBMs have distinct clinical features and survival differences. Under hypoxic conditions, the IDH gene is highly activated and mediates reductive glutamine to lipogenesis to maintain cell proliferation under hypoxia (39, 40). Because the gene expression profiles used in the study were derived from IDH^WT^ GBM tumor tissue samples, we did not observe significant differences in glucose metabolism in the risk groups defined by MRGPs. Similarly, most genes contained in the metabolic signature were related to redox, phosphorylation, and lipid metabolism. Increasing evidence indicates that metabolic rewiring in the tumor microenvironment may be responsible for changes in immune cell fate and function (41–44). Sören et al. (45) discovered that blood-derived TAMs but not microglia show altered metabolism and preferentially express immunosuppressive cytokines, which are associated with significantly poorer prognosis in glioma patients. In the present study, our signature also identified significant immune-related processes, such as phagocytosis regulation. Consistently, the abundance of monocytes in tumor tissue and the expression of some MRGs changed significantly with increasing risk levels. In the analysis of IDH^WT^ GBM single-cell RNA-seq data, we further determined that macrophage-related MRGs have closer molecular interactions with other MRGs than other cells, and identified 5 MRGs (ABCA1, MTHFD2, HMOX1, PIM1, and PTPRE) that were significantly changed in TAMs upon switching from low to high risk. Therefore, our study also provides a molecular profile integrating diverse biological processes to characterize the possible prognostic status of IDH^WT^ GBM patients.

The heterogeneity of TAMs has long been recognized as plasticity in response to different tumor microenvironments (46); however, the underlying mechanisms remain unclear. A recent study suggested that lipid accumulation and metabolism are required for TAM differentiation and activation (47). Our study identified that as tumors progressed, the expression of ABCA1 in TAMs was significantly increased, indicating that cholesterol metabolism plays a vital role in the functional polarization of TAMs. This finding is consistent with that of Goossens et al, who showed that ovarian cancer cells promote membrane cholesterol efflux in TAMs by upregulating ABCA1/G1 expression (48). Our and other studies have shown that cholesterol deletion can repolarize TAMs, promoting M2-to-M1 phenotypic conversion by downregulating ABCA1 expression (49). These results further confirm the reliability of our metabolic signature and may provide potential targets for IDH^WT^ GBM therapy.

Notably, although our findings indicate that the expression level of ABCA1 on TAMs can serve as a robust biomarker to assess the prognostic outcomes of GBMs, this study does have some limitations. In addition to ABCA1, other MRGs whose expression in TAMs increases significantly with increasing risk, alone or in combination, may also be more important in IDH^WT^ GBM development and progression. However, it is very challenging to characterize the protein expression levels of all prognostic MRGs in TAMs and to determine the weight of each MRG in prognostic stratification. Therefore, in this study, we selected only the highest-risk MRG ABCA1 for a biological proof-of-concept.

In summary, our MRGPs signature is a promising prognostic biomarker for individualized management of primary IDH^WT^ GBMs. Diverse biological processes involving metabolism and immunity in this study were integrated to outline a more complete molecular profile of the tumor.

## Data availability statement

The datasets presented in this study can be found in online repositories. The names of the repository/repositories and accession number(s) can be found in the article/**Supplementary Material**. The original data supporting the conclusions of this paper will be provided by the authors without undue retention.

## Ethics statement

The animal study was reviewed and approved by the Institutional Review Board of Nanjing University.

## Author contributions

All authors conceived and designed the study. SW, LL, and SZ contributed to collecting and analyzing the data. SW and LK performed the biological experiments. All authors participated in the writing of the manuscript. JW and JD conducted the critical revision of the manuscript. JW and JD provided financial support. All authors contributed to the article and approved the submitted version.

## Funding

This study was supported by the National Natural Science Foundation of China (82273261, 81773255 and 81472820).

## Acknowledgments

The authors thank all lab members and collaborators, especially Dr. Gang Meng, who contributed to this study. We also apologize to other researchers who contributed to this field but whose studies we did not discuss or cite due to limited space.

## Conflict of interest

The authors declare that the research was conducted in the absence of any commercial or financial relationships that could be construed as a potential conflict of interest.

## Publisher’s note

All claims expressed in this article are solely those of the authors and do not necessarily represent those of their affiliated organizations, or those of the publisher, the editors and the reviewers. Any product that may be evaluated in this article, or claim that may be made by its manufacturer, is not guaranteed or endorsed by the publisher.
