# Supplementary material for: Metabolic-related gene pairs signature analysis identifies ABCA1 expression levels on tumor-associated macrophages as a prognostic biomarker in primary IDHWT glioblastoma
Source: Front Immunol. 2022 Sep 30;13:869061. doi: 10.3389/fimmu.2022.869061 (PMC9561761; doi:10.3389/fimmu.2022.869061)

Supplementary Material

**Figure S1. Kaplan–Meier Curves of Overall Survival for Primary GBM With Different MRGP Risks in an Independent Validation Dataset**

**Figure S2. Kaplan–Meier Curves of Overall Survival for Upper and Bottom Quartiles of IDH^WT^ GBM With Different MRGP Risks**

**Figure S3. Kaplan–Meier Curves of Overall Survival for MGMTp Methylation and Unmethylated IDH^WT^ GBM With Different MRGP Risks**

**Figure S4. Kaplan–Meier Curves of Overall Survival for Classical, Mesenchymal, Neural, and Proneural IDH^WT^ GBM Subtypes With Different MRGP Risks**

**Figure S5. Prognostic Accuracy Comparison Between the Individualized MRGP Signature and Existing 9-gene Signature**

**Figure S6. Pearson Correlation Heatmap of 38 Signature Genes in Training and Validation Datasets**

**Figure S7. Necrosis Status of MRGP Risk Groups in TCGA Dataset**

**Figure S8. Pearson Correlation Heatmaps of the Expression of 38 MRGs and the Abundance of 22 Immune Cell types in Risk Groups**

**Figure S9. Defining the Risk Level of 28 Single-cell RNA Sequencing Samples Based on MRGPs Signature**

**Figure S10. Distribution of ABCA1 Expression in Tumor-infiltrating Immune Cell Subtypes**

**Figure S1. Kaplan–Meier Curves of Overall Survival for Primary GBM With Different MRGP Risks in an Independent Validation Dataset**

The GSE7696 dataset was used as an independent validation dataset. A total of 70 patients with primary GBM were stratified into high- and low-risk groups based on our MRGPs signature. The Kaplan–Meier survival curve of patients indicated that the survival of high-risk patients was worse than that of low-risk patients (*P* < 0.05, log-rank test).


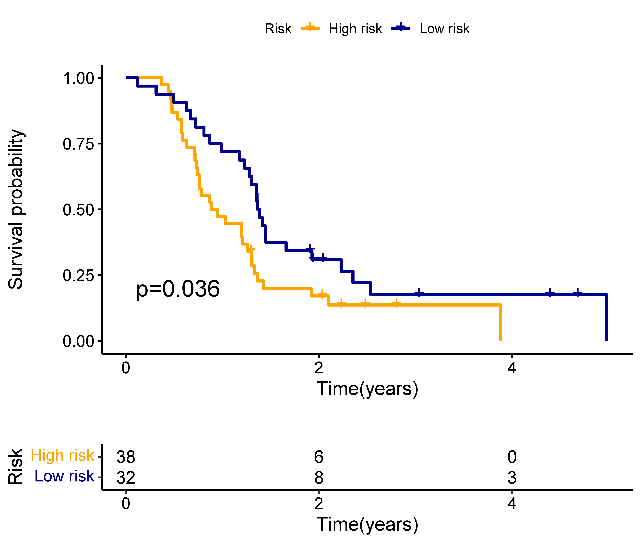


**Figure S2. Kaplan–Meier Curves of Overall Survival for Upper and Bottom Quartiles of IDH^WT^ GBM With Different MRGP Risks**

Patients in the training dataset were stratified into high- and low-risk groups based on the cut-off value. The Kaplan–Meier survival curve of patients with upper and lower quartile scores indicated that the survival of high-risk patients was worse than that of low-risk patients (*P* < 0.001, log-rank test).

**
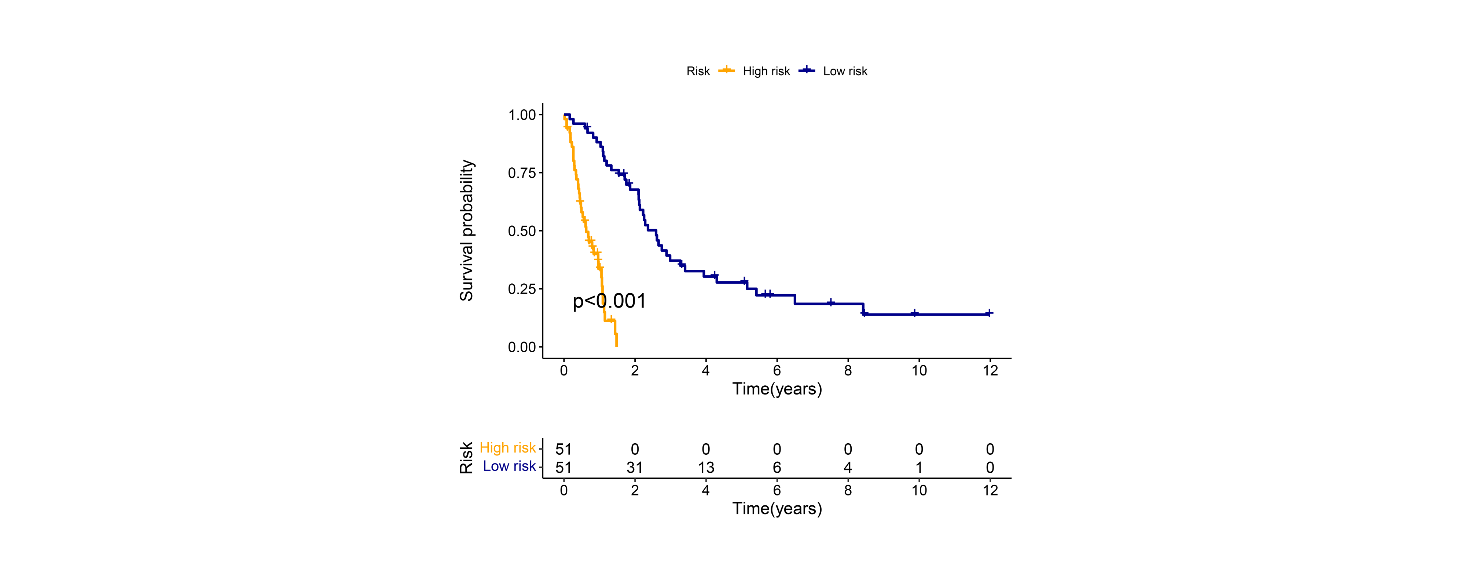
**

**Figure S3. Kaplan–Meier Curves of Overall Survival for MGMTp Methylation and Unmethylated IDH^WT^ GBM With Different MRGP Risks**

The overall survival of IDH^WT^ GBM with MGMTp methylation was stratified in training (**a**) and validation (**b**) datasets. The overall survival of IDH^WT^ GBM with MGMTp unmethylation was stratified in training (**c**) and validation (**d**) datasets. *P* values are all < 0.001. CI means C-index.


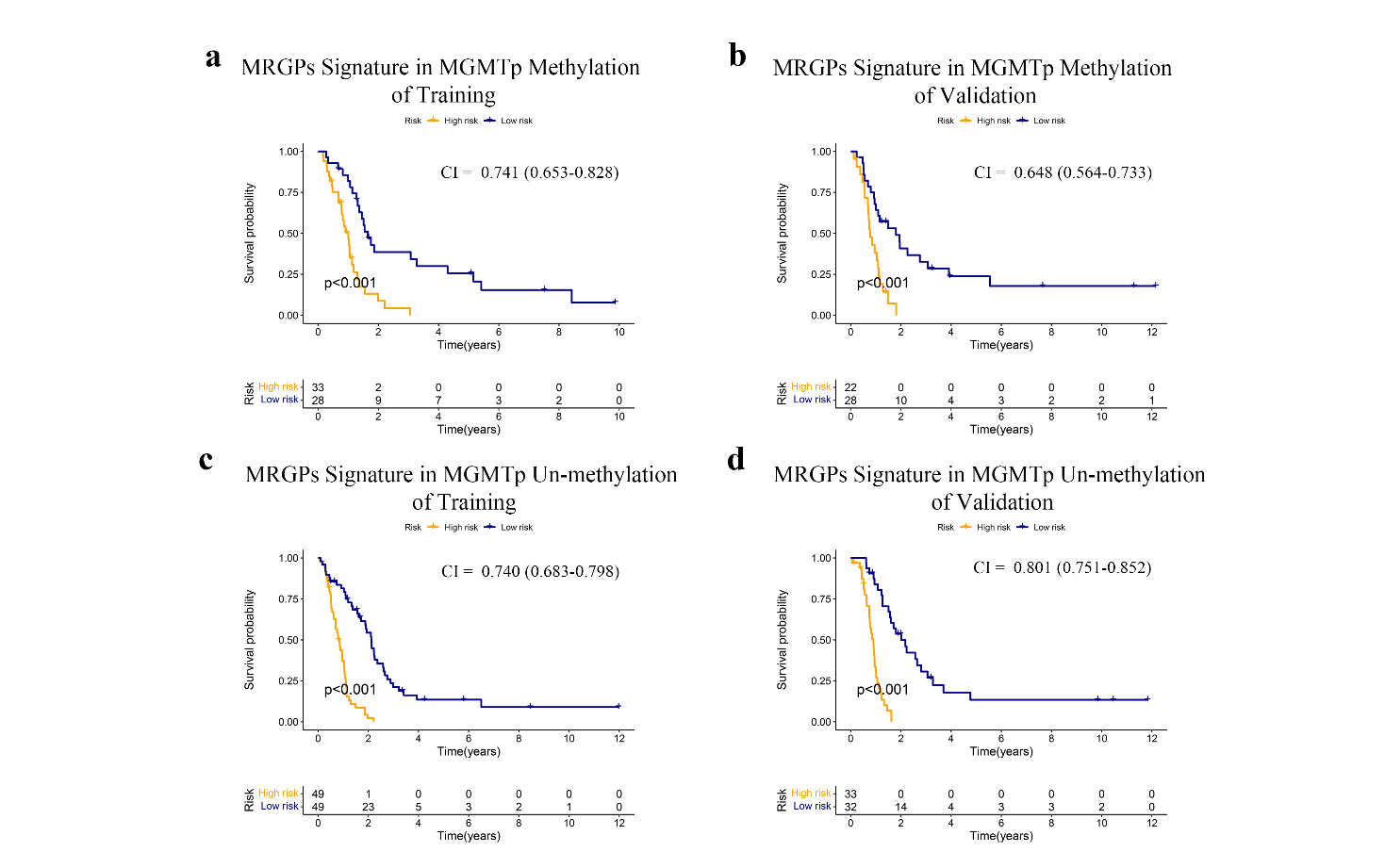


**Figure S4. Kaplan–Meier Curves of Overall Survival for** **Classical, Mesenchymal, Neural, and Proneural IDH^WT^ GBM Subtypes With Different MRGP Risks**

The overall survival of IDH^WT^ GBM with classical subtype was stratified in training (**a**) and validation (**b**) datasets. *P* values are all = 0.002. The overall survival of the mesenchymal subtype was stratified in training (**c**) and validation (**d**). *P* values are all < 0.001. The overall survival of the neural subtype was stratified in the training dataset (**e**, *P* = 0.041) but not in the validation dataset (**f**, *P* = 0.865). The overall survival of the proneural subtype was stratified in training (**g**, *P* = 0.032) and validation (**h**, *P* = 0.004). CI means C-index.


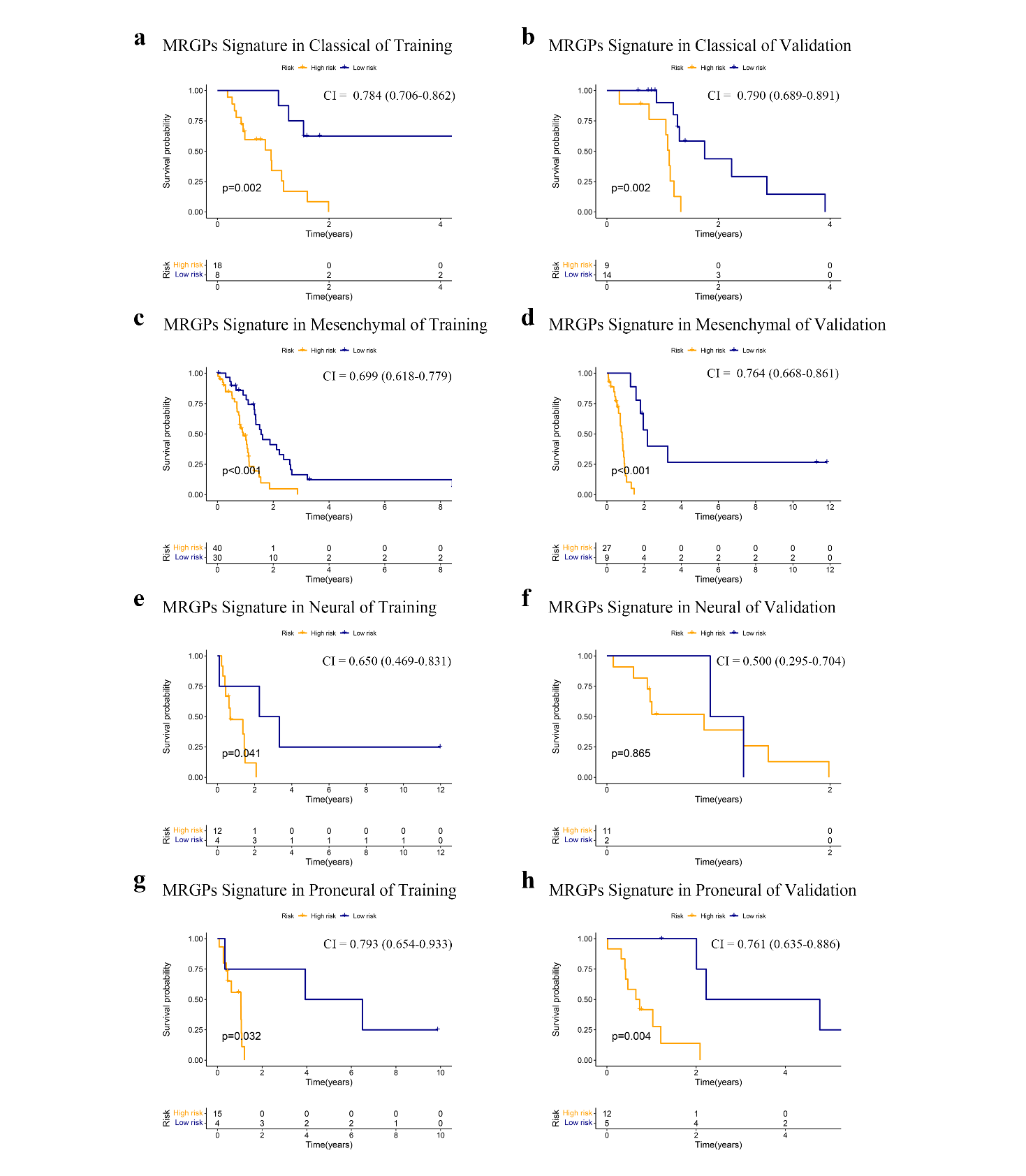


**Figure S5. Prognostic Accuracy Comparison Between the Individualized MRGP Signature and Existing 9-gene Signature**

The 9-gene expression matrix from the existing IDH^WT^ GBM signature was extracted for elastic net penalized Cox proportional hazards regression modeling in the training and validation datasets. **a**: Comparison of the C-index between our individualized MRGP signature and the existing 9-gene signature in the training and validation datasets. C-index = 0.5 indicates random prediction (white dashed line). 0.50 < C-index ≤ 0.70 means low accuracy. 0.70 < C-index ≤ 0.90 indicates medium accuracy. C-index > 0.90 means high accuracy. The bar represents the 95% confidence interval of the C-index. Kaplan–Meier curves of overall survival indicated the power of risk stratification of the 9-gene signature in the training (**b**) and validation (**c**) datasets.


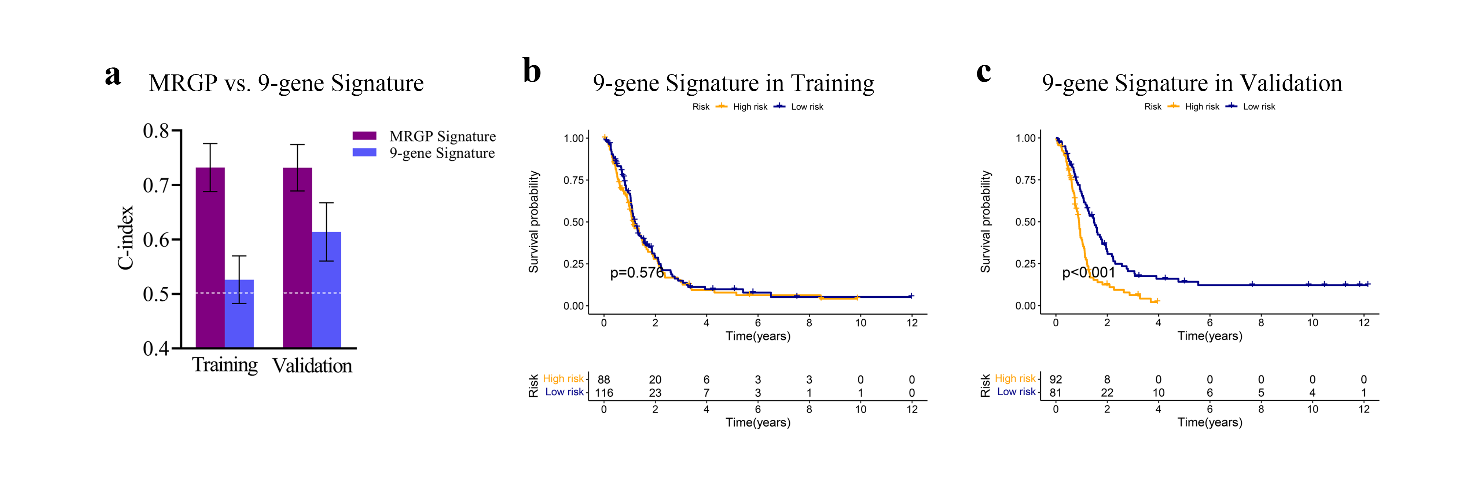


**Figure S6.** **Pearson Correlation Heatmap of 38 Signature Genes in Training and Validation Datasets**

The expression matrix of 38 signature genes was extracted for Pearson correlation analysis in the training (**a**) and validation (**b**) datasets. **P* < 0.05; ***P* < 0.01.


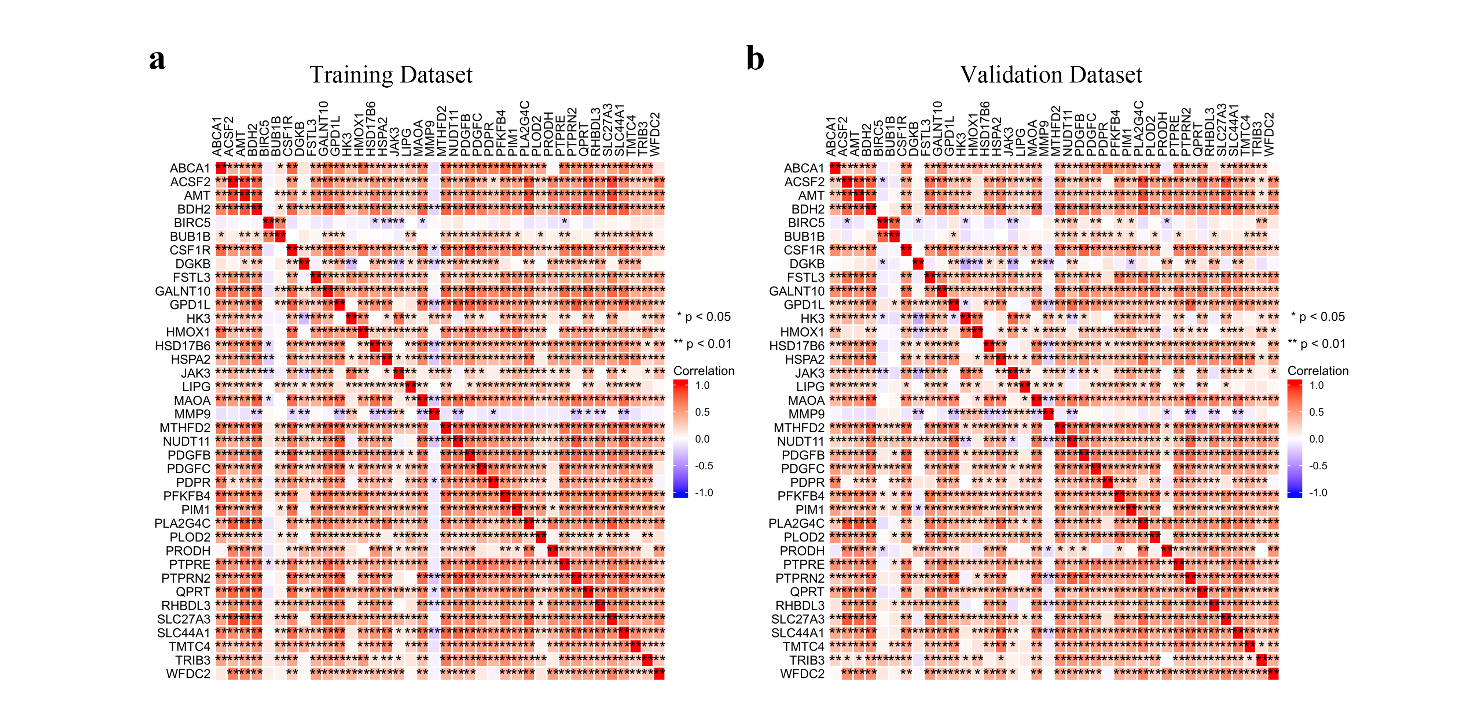


**Figure S7. Necrosis Status of MRGP Risk Groups in TCGA Dataset**

IDH^WT^ GBM patients from TCGA were divided into high- and low-risk groups. Sections from the top and bottom of the frozen tissue sample are shown. NS annotated as no significant difference between groups; * annotated as P < 0.05 (Mann–Whitney test).


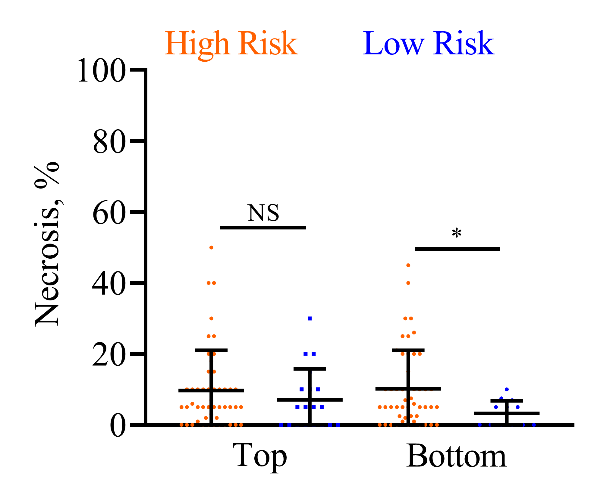


**Figure S8. Pearson Correlation Heatmaps of the Expression of 38 MRGs and the Abundance of 22 Immune Cell types in Risk Groups**

Patients from the meta dataset were stratified by high- and low-risk.


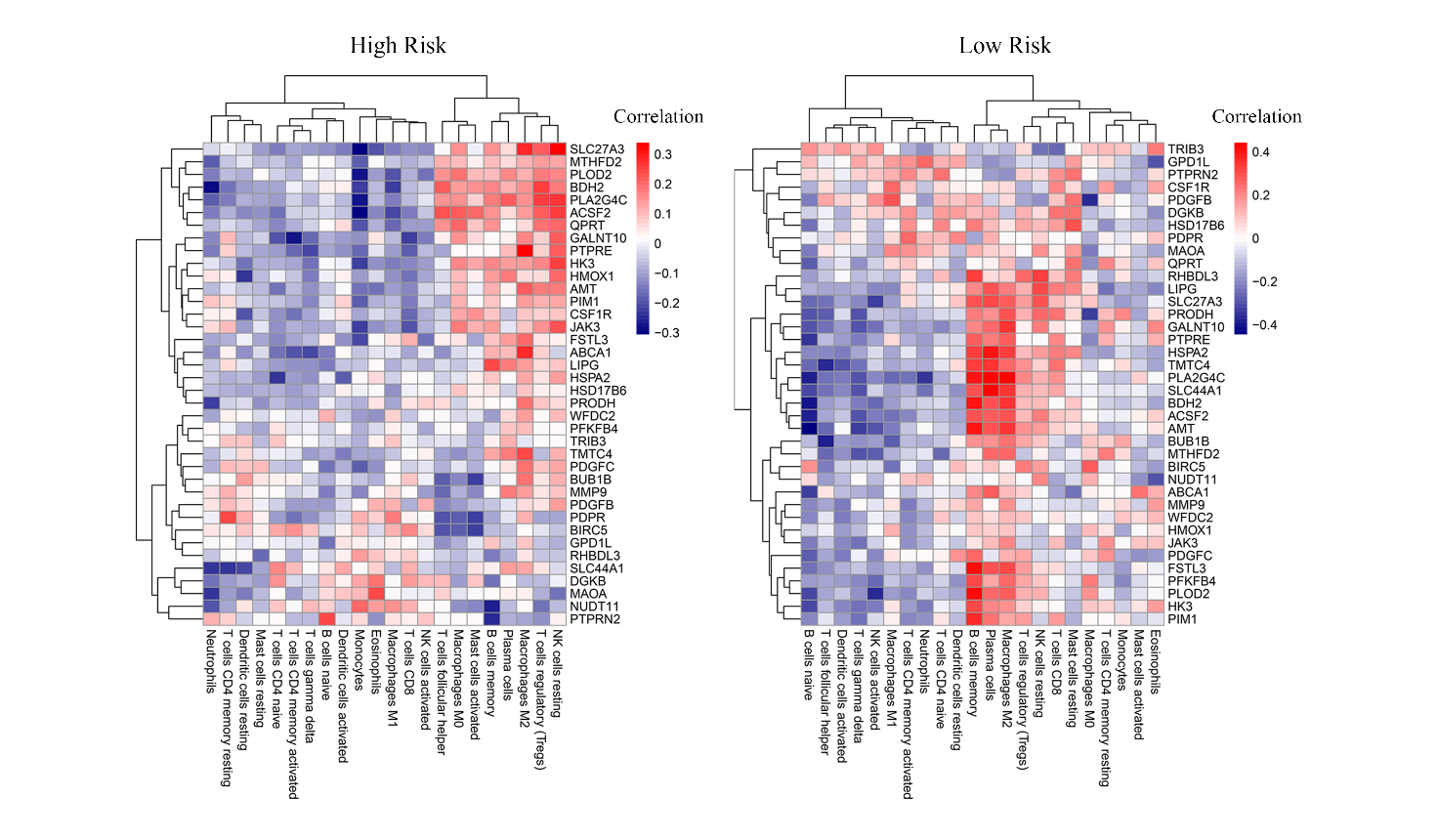


**Figure S9. Defining the Risk Level of 28 Single-cell RNA Sequencing Samples Based on MRGPs Signature**


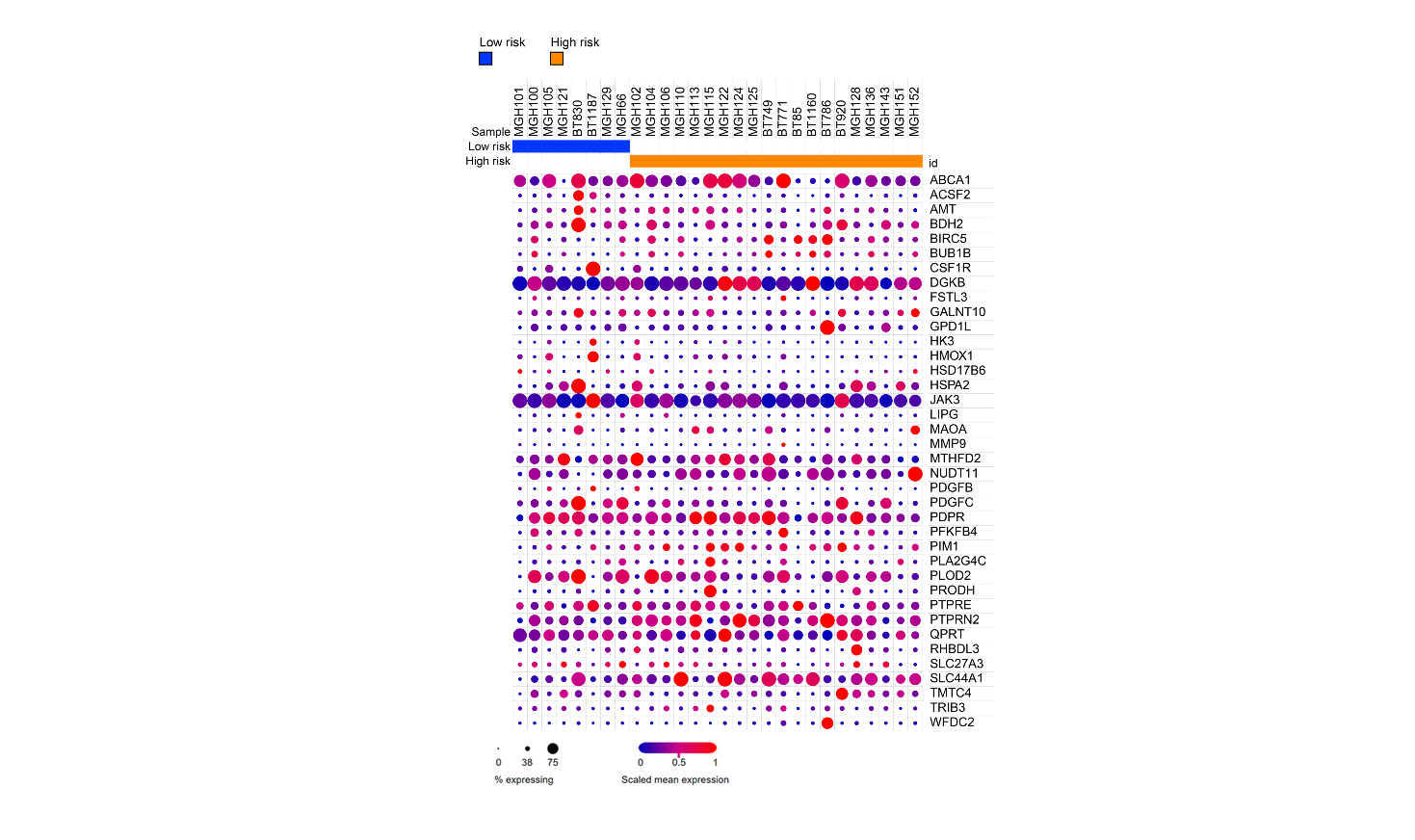


**Figure S10. Distribution of ABCA1 Expression in Tumor-infiltrating Immune Cell Subtypes**

C57 mice were intracranially inoculated with GL261 cells. On day 14, mice were sacrificed and collected tumor tissues. The expression levels of ABCA1 in tumor cells and infiltrating immune cell subsets were detected by flow cytometry (n, 3 or 6). FMO indicates fluorescence minus one. The difference between risk groups was calculated by the student-t test (*P < 0.05, **P < 0.01, ***P < 0.001).


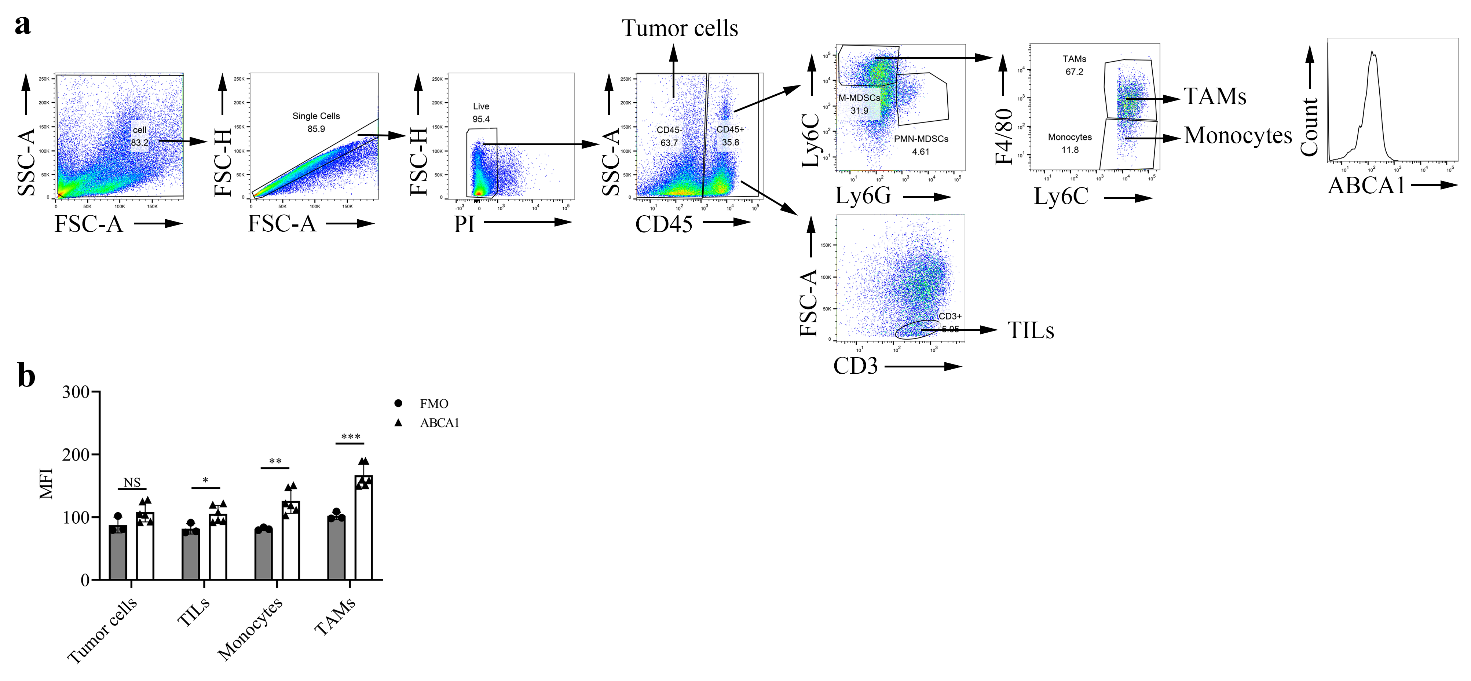

Supplement: Supplementary file 1 [file DataSheet_1.docx]
