# Supplementary material for: Metabolic-related gene pairs signature analysis identifies ABCA1 expression levels on tumor-associated macrophages as a prognostic biomarker in primary IDHWT glioblastoma
Source: Front Immunol. 2022 Sep 30;13:869061. doi: 10.3389/fimmu.2022.869061 (PMC9561761; doi:10.3389/fimmu.2022.869061)
Supplement: Supplementary file 2 [file DataSheet_2.docx]

Supplementary Material

**Supplemental Methods**

**Supplemental Results**

**Supplemental References**

**Table S1. Details About the Datasets Used in This Study**

**Table S2. Clinical Characteristics of Patients in the Training and Validation Datasets**

**Table S3. List of KEGG Metabolic Pathways Used in This Study**

**Table S4. Details of 21 MRGPs to Construct the Prognostic Model**

**Table S5. Univariate and Multivariate Analyses of Prognostic Factors in Training and Testing Datasets**

**Table S6. GO Biological Processes Represented by 38 MRGs That Constitute the Prognostic Model**

**Table S7. Real-Time PCR Primer Sequences**

**Supplemental Methods**

***Data Preprocessing***

For Illumina HiSeq datasets, TCGA processed FPKM files were downloaded; CGGA FPKM files further proceeded to log2 transformation. For Aligent microarray data, probe intensity values were normalized by using GeneSpring GX11.0.(1) Probes targeting multiple genes were removed. If multiple probe sets target the same gene, the average signal was defined as its expression level. Ensembl IDs were converted to official gene symbols. To explore biologically preferential transcription, we removed genes with relatively low variation (median absolute deviation [MAD] < 0.5).(2)

We collected 3679 metabolic-related genes (MRGs) from 89 metabolism-related pathways (Table S3) in the Kyoto Encyclopedia of Genes and Genomes (KEGG) database (http://www.kegg.jp/).(3) Only MRGs shared in all datasets were retained and further merged into a meta dataset.

Subsequently, we utilized the “for” function to perform a pair comparison of 802 common MRGs in each sample to generate MRG pairs (MRGPs). Briefly, the score was 1 if MRG 1 was larger than MRG 2; otherwise, it was 0. A total of 2660 MRGPs (1.97%) were to have constant ordering (all o or 1) in the TCGA or CGGA dataset, which may be caused by platform bias. To reduce cross-platform batch effects, we removed MRGPs with low variation (MAD = 0) and selected MRGPs with a ratio between 0.2 and 0.8 in the overall sample number as candidates for subsequent analysis.(4) Finally, a new “0, 1” MRGPs matrix was generated. Gene expression and clinical annotation data were collected from May through October 2021.

***Robustness Assessment of the 21 MRGPs***

In the training cohort, we repeated feature selection and modeling construction 1,000 times to evaluate the robustness of the 21 MRGPs. Those MRGPs that appear in at least one model were selected to calculate a background distribution of frequencies. The frequencies of the 21 MRGPs consisting of the prognostic model were compared with the background distribution by using the Mann–Whitney test.

***Comparison with Existing 9-Gene Signature***

The development of the existing 9-gene IDH^WT^ GBM signature (GSE150615) was based on the NanoString gene expression data of FFPE samples, including CHEK1, GPR17, IGF2BP3, MGMT, MTHFD1L, PTRH2, SOX11, S100A9, and TFRC.(5) Briefly, the gene expression values of the 9 prognostic genes were selected in the transformed meta dataset, and the stratification of survival risk for each patient was based on elastic net penalized Cox proportional hazards regression (*P* < 0.05). The C-index of the MRGPs signature and 9-gene signature was calculated in training and validation to assess their performance variations.

***RNA Isolation and Real-Time PCR***

Total RNA from cultured cells was extracted by using TRIzol reagent (Cat No. 15596-026, Invitrogen) and was reverse-transcribed by using PrimeScriptTM RT Master Mix (Cat No. RR036A, TaKaRa). Real-time PCR was performed by using AceQ Universal SYBR qPCR Master Mix (Cat No. Q511-02, Vazyme) on a ViiA 7 Real-Time PCR System (Applied Biosystems). Each reaction was performed in triplicate. Gene expression was calculated by the comparative CT method and normalized to that of GAPDH. All primer sequences used for real-time PCR are listed in Table S7.

***Determination of IDH1 Mutation Status of Human GBM Cell Lines***

The genomic DNA of GBM cell lines was extracted for PCR cloning of the IDH1 gene. The hIDH1 forward primer sequence was CTATGATTTAGGCATAGAGAA GBM cells; the hIDH1 reverse primer sequence was TTGATCCCCATAAGCATG. The PCR products were sequenced to identify gene mutations. Figure 2e shows that the GBM cell lines used in this study were all IDH1 wild-type.

***Immunohistochemistry***

To detect the expression of ABCA1, coronal paraffin sections of brain tissue from GL261^IDH-WT^ tumor-bearing mice on day 21 were used for immunohistochemical assays. The following antibodies were used: anti-mouse/human ABCA1 rabbit polyclonal antibody (Sangon Biotech, Cat No. 14427-1-AP, 1:100 dilution) and HRP-conjugated goat anti-rabbit IgG (Servicebio, Cat No. D110058-0100, 1:200 dilution).

**Supplemental Results**

***Robustness of the 21 MRGPs***

Cycle modeling identified that a total of 38 MRGPs appeared in at least 1 model (frequency distribution from 1.23% to 100%). Compared with the background distribution of frequencies for all 38 MRGPs, our 21 MRGPs constituting the prognostic model possessed higher frequencies (*P* < 0.001). The smallest frequency of our 21 MRGPs was 61.7% (ranked in the top 50% of that for 38 MRGPs). The frequency of 20 out of our 21 MRGPs ranked in the top 25% of all 38 MRGPs. The 13 highest frequency MRGPs among 38 MRGPs were all included in our 21 MGRPs. Therefore, we confirmed that the MRGP signature is robust compared with randomizations. The frequency distribution diagram as shown below. Blue bars represent our 21 MRGPs.


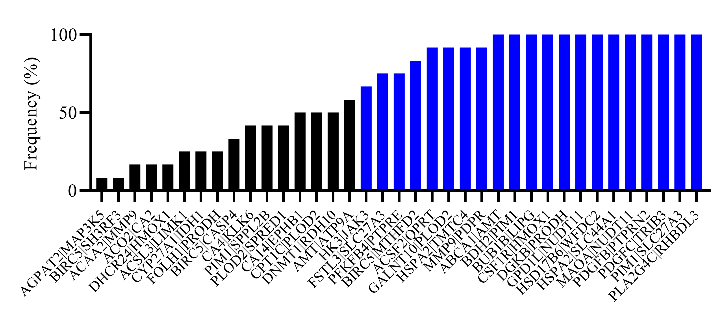


**Supplemental References**

1. Zhao Z, Zhang KN, Wang Q, Li G, Zeng F, Zhang Y, et al. Chinese Glioma Genome Atlas (CGGA): A Comprehensive Resource with Functional Genomic Data from Chinese Glioma Patients. *Genomics Proteomics Bioinformatics* (2021) 19(1):1-12. Epub 2021/03/05. doi: 10.1016/j.gpb.2020.10.005.

2. Guinney J, Dienstmann R, Wang X, de Reynies A, Schlicker A, Soneson C, et al. The consensus molecular subtypes of colorectal cancer. *Nat Med* (2015) 21(11):1350-6. Epub 2015/10/13. doi: 10.1038/nm.3967.

3. Kanehisa M, Sato Y, Kawashima M, Furumichi M, Tanabe M. KEGG as a reference resource for gene and protein annotation. *Nucleic Acids Res* (2016) 44(D1):D457-62. Epub 2015/10/18. doi: 10.1093/nar/gkv1070.

4. Leek JT, Scharpf RB, Bravo HC, Simcha D, Langmead B, Johnson WE, et al. Tackling the widespread and critical impact of batch effects in high-throughput data. *Nat Rev Genet* (2010) 11(10):733-9. Epub 2010/09/15. doi: 10.1038/nrg2825.

5. Johnson RM, Phillips HS, Bais C, Brennan CW, Cloughesy TF, Daemen A, et al. Development of a gene expression-based prognostic signature for IDH wild-type glioblastoma. *Neuro Oncol* (2020) 22(12):1742-56. Epub 2020/09/09. doi: 10.1093/neuonc/noaa157.

**Table S1. Details About the Datasets Used in This Study**

| **Dataset ID** | **Platform** | **Normalization** | **No. of Patients** |
| --- | --- | --- | --- |
| TCGA GBM | Illumina HiSeq | Download normalized FPKM from TCGA GDC | 129 |
| CGGA_325 (mRNAseq_325) | Illumina HiSeq | Download normalized FPKM from CGGA | 105 |
| CGGA_693 (mRNAseq_693) | Illumina HiSeq | Download normalized FPKM from CGGA | 74 |
| CGGA_301 (mRNA-array_301) | Agilent Microarray | Probe intensity normalization using GeneSpring GX11.0 | 69 |
| GSE7696 | Affymetrix Human Genome U133 Plus 2.0 Array | Download normalized RMA from GEO | 70 |

**Table S2. Clinical Characteristics of Patients in the Training and Validation Datasets**

|  | **Subgroups** | **Training** | **Validation** | ***P* value^*^** | **TCGA** | **CGGA_325** | **CGGA_693** | **CGGA_301** |
| --- | --- | --- | --- | --- | --- | --- | --- | --- |
| **No. of samples** |  | 204 | 173 |  | 129 | 74 | 105 | 69 |
| **Median age in years (range)** |  | 54 (12-89) | 58 (11-85) | 0.138 | 62 (24-89) | 54 (11-79) | 56 (14-76) | 50 (12-70) |
| **Gender (%)** | Female  Male | 129 (63)  75 (37) | 107 (62)  66 (38) | 0.831 | 85 (66)  44 (34) | 48 (65)  26 (35) | 61 (58)  44 (42) | 42 (61)  27 (39) |
| **Subtypes (%)** | Classical  Mesenchymal  Neural  Proneural | 22 (11)  53 (26)  16 (8)  17 (8) | 23 (13)  35 (20)  13 (8)  18 (10) | 0.525 | 34 (26)  43 (33)  24 (19)  27 (21) | NA | NA | 11 (16)  45 (65)  5 (7)  8 (12) |
| **1p19q codeletion** | Yes  No | 1 (1)  146 (71) | 1 (1)  135 (78) | 0.956 | 0 (0)  126 (98) | 0 (0)  73 (99) | 2 (2)  82 (78) | NA |
| **MGMTp methylation** | Yes  No | 61 (30)  98 (48) | 50 (29)  65 (38) | 0.395 | 15 (12)  26 (20) | 23 (31)  49 (66) | 52 (50)  40 (38) | 21 (30)  48 (70) |
| **Radiation therapy** | Yes  No | 169 (83)  23 (11) | 134 (77)  26 (15) | 0.249 | 103 (80)  17 (13) | 57 (77)  8 (11) | 89 (85)  11 (10) | 54 (78)  13 (19) |
| **Chemical therapy (TMZ)** | Yes  No | 136 (67)  54 (26) | 116 (67)  41 (24) | 0.632 | 101 (78)  18 (14) | 45 (61)  18 (24) | 59 (56)  42 (40) | 47 (68)  17 (25) |
| **No. of death (%)** |  | 162 (80) | 141 (81) | 0.395 | 86 (67) | 64 (86) | 92 (88) | 61 (88) |
| **Median follow-up in years** |  | 1.06 | 0.95 |  | 0.76 | 1.09 | 1.12 | 1.13 |

Abbreviations: TCGA, TCGA glioblastoma dataset; CGGA, CGGA glioblastoma dataset; NA, unavailable; MGMTp; O-6-methylguanine-DNA methyltransferase promoter; TMZ, temozolomide.

^*^ annotated as the difference between training and validation datasets in terms of clinical factors. Age was compared using the Mann–Whitney test. Subtype, 1p19q codeletion, MGMT methylation, radiation therapy, and chemical therapy were compared using the chi-squared test. The log-rank (Mantel–Cox) test was used to assess differences in follow-up data.

**Table S3. List of KEGG Metabolic Pathways Used in This Study**

| **Entry** | **Term** | **Entry** | **Term** |
| --- | --- | --- | --- |
| hsa00010 | Glycolysis / Gluconeogenesis | hsa00515 | Mannose type O-glycan biosynthesis |
| hsa00020 | Citrate cycle (TCA cycle) | hsa00520 | Amino sugar and nucleotide sugar metabolism |
| hsa00030 | Pentose phosphate pathway | hsa00524 | Neomycin, kanamycin and gentamicin biosynthesis |
| hsa00040 | Pentose and glucuronate interconversions | hsa00531 | Glycosaminoglycan degradation |
| hsa00051 | Fructose and mannose metabolism | hsa00532 | Glycosaminoglycan biosynthesis - chondroitin sulfate / dermatan sulfate |
| hsa00052 | Galactose metabolism | hsa00533 | Glycosaminoglycan biosynthesis - keratan sulfate |
| hsa00053 | Ascorbate and aldarate metabolism | hsa00534 | Glycosaminoglycan biosynthesis - heparan sulfate / heparin |
| hsa00061 | Fatty acid biosynthesis | hsa00561 | Glycerolipid metabolism |
| hsa00062 | Fatty acid elongation | hsa00562 | Inositol phosphate metabolism |
| hsa00071 | Fatty acid degradation | hsa00563 | Glycosylphosphatidylinositol (GPI)-anchor biosynthesis |
| hsa00072 | Synthesis and degradation of ketone bodies | hsa00564 | Glycerophospholipid metabolism |
| hsa00100 | Steroid biosynthesis | hsa00565 | Ether lipid metabolism |
| hsa00120 | Primary bile acid biosynthesis | hsa00590 | Arachidonic acid metabolism |
| hsa00130 | Ubiquinone and other terpenoid-quinone biosynthesis | hsa00591 | Linoleic acid metabolism |
| hsa00140 | Steroid hormone biosynthesis | hsa00592 | alpha-Linolenic acid metabolism |
| hsa00190 | Oxidative phosphorylation | hsa00600 | Sphingolipid metabolism |
| hsa00220 | Arginine biosynthesis | hsa00601 | Glycosphingolipid biosynthesis - lacto and neolacto series |
| hsa00230 | Purine metabolism | hsa00603 | Glycosphingolipid biosynthesis - globo and isoglobo series |
| hsa00232 | Caffeine metabolism | hsa00604 | Glycosphingolipid biosynthesis - ganglio series |
| hsa00240 | Pyrimidine metabolism | hsa00620 | Pyruvate metabolism |
| hsa00250 | Alanine, aspartate and glutamate metabolism | hsa00630 | Glyoxylate and dicarboxylate metabolism |
| hsa00260 | Glycine, serine and threonine metabolism | hsa00640 | Propanoate metabolism |
| hsa00270 | Cysteine and methionine metabolism | hsa00650 | Butanoate metabolism |
| hsa00280 | Valine, leucine and isoleucine degradation | hsa00670 | One carbon pool by folate |
| hsa00290 | Valine, leucine and isoleucine biosynthesis | hsa00730 | Thiamine metabolism |
| hsa00310 | Lysine degradation | hsa00740 | Riboflavin metabolism |
| hsa00330 | Arginine and proline metabolism | hsa00750 | Vitamin B6 metabolism |
| hsa00340 | Histidine metabolism | hsa00760 | Nicotinate and nicotinamide metabolism |
| hsa00350 | Tyrosine metabolism | hsa00770 | Pantothenate and CoA biosynthesis |
| hsa00360 | Phenylalanine metabolism | hsa00780 | Biotin metabolism |
| hsa00380 | Tryptophan metabolism | hsa00785 | Lipoic acid metabolism |
| hsa00400 | Phenylalanine, tyrosine and tryptophan biosynthesis | hsa00790 | Folate biosynthesis |
| hsa00410 | beta-Alanine metabolism | hsa00830 | Retinol metabolism |
| hsa00430 | Taurine and hypotaurine metabolism | hsa00860 | Porphyrin and chlorophyll metabolism |
| hsa00440 | Phosphonate and phosphinate metabolism | hsa00900 | Terpenoid backbone biosynthesis |
| hsa00450 | Selenocompound metabolism | hsa00910 | Nitrogen metabolism |
| hsa00471 | D-Glutamine and D-glutamate metabolism | hsa00920 | Sulfur metabolism |
| hsa00472 | D-Arginine and D-ornithine metabolism | hsa00980 | Metabolism of xenobiotics by cytochrome P450 |
| hsa00480 | Glutathione metabolism | hsa00982 | Drug metabolism - cytochrome P450 |
| hsa00500 | Starch and sucrose metabolism | hsa00983 | Drug metabolism - other enzymes |
| hsa00510 | N-Glycan biosynthesis | hsa01040 | Biosynthesis of unsaturated fatty acids |
| hsa00511 | Other glycan degradation | hsa05230 | Central carbon metabolism in cancer |
| hsa00512 | Mucin type O-glycan biosynthesis | hsa05231 | Choline metabolism in cancer |
| hsa00513 | Various types of N-glycan biosynthesis | hsa04979 | Cholesterol metabolism |
| hsa00514 | Other types of O-glycan biosynthesis |  |  |
|  |  |  |  |

**Table S4. Details of 21 MRGPs to Construct the Prognostic Model**

| **No.** | **MRG 1** \| **MRG2** | **Full name 1** \| **Full name 2** | **Coefficient** |
| --- | --- | --- | --- |
| MRGP-01 | ABCA1 \| AMT | ATP Binding Cassette Subfamily A Member 1 \| Aminomethyltransferase | 0.219603483 |
| MRGP-02 | PIM1 \| SLC27A3 | Pim-1 Proto-Oncogene, Serine/Threonine Kinase \| Solute Carrier Family 27 Member 3 | 0.143993898 |
| MRGP-03 | BUB1B \| LIPG | BUB1 Mitotic Checkpoint Serine/Threonine Kinase B \| Lipase G, Endothelial Type | 0.06603579 |
| MRGP-04 | DGKB \| PRODH | Diacylglycerol Kinase Beta \| Proline Dehydrogenase 1 | 0.061380657 |
| MRGP-05 | MMP9 \| PDPR | Matrix Metallopeptidase 9 \| Pyruvate Dehydrogenase Phosphatase Regulatory Subunit | 0.056867035 |
| MRGP-06 | FSTL3 \| SLC27A3 | Follistatin Like 3 \| Solute Carrier Family 27 Member 3 | 0.032096551 |
| MRGP-07 | BIRC5 \| MTHFD2 | Baculoviral IAP Repeat Containing 5 \| Methylenetetrahydrofolate Dehydrogenase (NADP+ Dependent) 2 | 0.02486413 |
| MRGP-08 | PFKFB4 \| PTPRE | 6-Phosphofructo-2-Kinase/Fructose-2,6-Biphosphatase 4 \| Protein Tyrosine Phosphatase Receptor Type E | 0.008831711 |
| MRGP-09 | HK3 \| JAK3 | Hexokinase 3 \| Janus Kinase 3 | 0.006743194 |
| MRGP-10 | GALNT10 \| PLOD2 | Polypeptide N-Acetylgalactosaminyltransferase 10 \| Procollagen-Lysine,2-Oxoglutarate 5-Dioxygenase 2 | -0.021439955 |
| MRGP-11 | CSF1R \| HMOX1 | Colony Stimulating Factor 1 Receptor \| Heme Oxygenase 1 | -0.021823545 |
| MRGP-12 | HSPA2 \| TMTC4 | Heat Shock Protein Family A (Hsp70) Member 2 \| Transmembrane O-Mannosyltransferase Targeting Cadherins 4 | -0.025687787 |
| MRGP-13 | ACSF2 \| QPRT | Acyl-CoA Synthetase Family Member 2 \| Quinolinate Phosphoribosyltransferase | -0.032907908 |
| MRGP-14 | HSPA2 \| SLC44A1 | Heat Shock Protein Family A (Hsp70) Member 2 \| Solute Carrier Family 44 Member 1 | -0.0508548 |
| MRGP-15 | GPD1L \| NUDT11 | Glycerol-3-Phosphate Dehydrogenase 1 Like \| Nudix Hydrolase 11 | -0.073007795 |
| MRGP-16 | BDH2 \| PIM1 | 3-Hydroxybutyrate Dehydrogenase 2 \| Pim-1 Proto-Oncogene, Serine/Threonine Kinase | -0.093948686 |
| MRGP-17 | MAOA \| NUDT11 | Monoamine Oxidase A \| Nudix Hydrolase 11 | -0.095702873 |
| MRGP-18 | PLA2G4C \| RHBDL3 | Phospholipase A2 Group IVC \| Rhomboid Like 3 | -0.153422196 |
| MRGP-19 | PDGFB \| PTPRN2 | Platelet Derived Growth Factor Subunit B \| Protein Tyrosine Phosphatase Receptor Type N2 | -0.15889971 |
| MRGP-20 | PDGFC \| TRIB3 | Platelet Derived Growth Factor C \| Tribbles Pseudokinase 3 | -0.181147198 |
| MRGP-21 | HSD17B6 \| WFDC2 | Hydroxysteroid 17-Beta Dehydrogenase 6 \| WAP Four-Disulfide Core Domain 2 | -0.214267192 |

**Table S5. Univariate and Multivariate Analyses of Prognostic Factors in Training and Testing Datasets**

| **Datasets** |  | **Univariate** | | **Multivariate** | |
| --- | --- | --- | --- | --- | --- |
|  | **Variable** | **Hazard ratio (95% CI)** | ***P* value^a^** | **Hazard ratio (95% CI)** | ***P* value^b^** |
| **Training** | **Age** | 1.019 (1.005-1.033) | 0.009 | 0.999 (0.984-1.015) | 0.930 |
|  | **Gender** | 1.032 (0.730-1.459) | 0.858 | 0.972 (0.680-1.388) | 0.874 |
|  | **MGMTp methylation** | 1.118 (0.786-1.591) | 0.535 | 1.121 (0.783-1.603) | 0.533 |
|  | **Metabolic risk scrore** | 6.676 (4.526-9.849) | < 0.001 | 6.698 (4.478-10.018) | < 0.001 |
| **Validation** | **Age** | 1.021 (1.005-1.037) | 0.010 | 1.013 (0.996-1.030) | 0.140 |
|  | **Gender** | 1.069 (0.709-1.613) | 0.750 | 1.167 (0.752-1.812) | 0.490 |
|  | **MGMTp methylation** | 1.009 (0.672-1.516) | 0.966 | 1.093 (0.715-1.673) | 0.681 |
|  | **Metabolic risk scrore** | 5.921 (3.703-9.470) | < 0.001 | 5.714 (3.542-9.218) | < 0.001 |

Age, gender, and MGMTp methylation status were coded as continuous variables.

^a^ *P* value was calculated by using the log-rank (Mantel–Cox) test.

^b^ *P* value was calculated by using the Wald test of the Cox proportional hazard regression model.

**Table S6.** **GO Biological Processes Represented by 38 MRGs That Constitute the Prognostic Model**

| **GO ID** | **Term** | ***P* value** | **Genes** |
| --- | --- | --- | --- |
| GO:0055114 | oxidation-reduction process | 0.001479 | PDPR, MAOA, MTHFD2, PLOD2, GPD1L, HSD17B6, PRODH |
| GO:0006468 | protein phosphorylation | 0.002719 | PDGFB, PIM1, BIRC5, BUB1B, TRIB3, JAK3 |
| GO:0055091 | phospholipid homeostasis | 0.019135 | ABCA1, LIPG |
| GO:0019674 | NAD metabolic process | 0.023338 | QPRT, GPD1L |
| GO:0009395 | phospholipid catabolic process | 0.029611 | LIPG, PLA2G4C |
| GO:1904707 | positive regulation of vascular smooth muscle cell proliferation | 0.031693 | PDGFB, MMP9 |
| GO:0030225 | macrophage differentiation | 0.037913 | CSF1R, MMP9 |
| GO:0043691 | reverse cholesterol transport | 0.037913 | ABCA1, LIPG |
| GO:0018108 | peptidyl-tyrosine phosphorylation | 0.042458 | CSF1R, PDGFB, JAK3 |
| GO:0008283 | cell proliferation | 0.043169 | CSF1R, LIPG, PIM1, BUB1B |
| GO:0031954 | positive regulation of protein autophosphorylation | 0.044095 | PDGFC, PDGFB |
| GO:0046835 | carbohydrate phosphorylation | 0.048194 | HK3, PFKFB4 |

**Table S7. Real-Time PCR Primer Sequences**

| **Locus** | **Forward** | **Reverse** |
| --- | --- | --- |
| GAPDH | GTCTCCTCTGACTTCAACAGCG | ACCACCCTGTTGCTGTAGCCAA |
| ABCA1 | CAGGCTACTACCTGACCTTGGT | CTGCTCTGAGAAACACTGTCCTC |
| ACSF2 | CTCCAGAGTTGATCCGAGCCAT | CTTCTGCTCCACAGTGTCCTCA |
| AMT | GTGGTTGGAGACATTGCAGAGC | AGCGTTGGACACCACATACAGG |
| BDH2 | CACAACCAAGGCAGCCGTGATT | CTTGTAGAGATGGCGTATCAACTG |
| BIRC5 | CCACTGAGAACGAGCCAGACTT | GTATTACAGGCGTAAGCCACCG |
| BUB1B | GTGGAAGAGACTGCACAACAGC | TCAGACGCTTGCTGATGGCTCT |
| CSF1R | GCTGCCTTACAACGAGAAGTGG | CATCCTCCTTGCCCAGACCAAA |
| DGKB | CCTGACTTCAGAGTGTTAGCCTG | CAAGAGGCAGAATCGCAACTGG |
| FSTL3 | ACATTGACACCGCCTGGTCCAA | ACTCCACGCCGTCGCACGAAT |
| GALNT10 | GAGCCTTTGACTGGGAGATGTAC | GAGTTCCCAGAACCACTTCCGA |
| GPD1L | CCGTGGTTGATGATGCAGACAC | CGCTTTGGTGTTGTCTCCACAG |
| HK3 | CATCGTGGACTTCCAGCAGAAG | CTTGGTCCAGTTCAGGAGGATG |
| HMOX1 | CCAGGCAGAGAATGCTGAGTTC | AAGACTGGGCTCTCCTTGTTGC |
| HSD17B6 | CCAGCATTCTGGGAAGAGTTGC | CCGTTCTGAAGTAGCCAGGTTC |
| HSPA2 | GACCAAGGACAATAACCTGCTGG | GGCGTCAATGTCGAAGGTAACC |
| JAK3 | AGTGACCCTCACTTCCTGCTGT | GGCTGAACCAAGGATGATGTGG |
| LIPG | CTGTGGACTCAACGATGTCTTGG | ACTCGGCTTGTCCTGATTCACC |
| MAOA | TCTGAGCCTCACGAAGTGTCTG | ATCCGTTCGCTCACTTGACCAG |
| MMP9 | GCCACTACTGTGCCTTTGAGTC | CCCTCAGAGAATCGCCAGTACT |
| MTHFD2 | CTCCTTGTTCAGTTGCCTCTTCC | CTGATCCAAACACATTCGTCCTAC |
| NUDT11 | ACAGAACCAGGATCGCAAGCAC | GCACTGGAGAACCTTGATGGCA |
| PDGFB | GAGATGCTGAGTGACCACTCGA | GTCATGTTCAGGTCCAACTCGG |
| PDGFC | TGAACCAGGGTTCTGCATCCAC | TAAGCAGGTCCAGTGGCAAAGC |
| PDPR | TGCCGAAAGACAGCAACCTGCT | GGAAGTGGTCTGGAGTCATAGG |
| PFKFB4 | GATCCTGAGGTCATAGCTGCCA | CTATCCAGGTCCTCATCTAGCG |
| PIM1 | TCTACTCAGGCATCCGCGTCTC | CTTCAGCAGGACCACTTCCATG |
| PLA2G4C | GGAAGACTGGTCAGAACTCACC | GCATTAGCAACAGCCCTTCTCC |
| PLOD2 | GACAGCGTTCTCTTCGTCCTCA | CTCCAGCCTTTTCGTGGTGACT |
| PRODH | AGAGTCAGCGATGACGGCTTCA | CTTGCTCCACAGCCATTTGGTG |
| PTPRE | TGATTGACCTCATCGCAGCCGT | CTCGCTCCAAAATGTTGCTGAGG |
| PTPRN2 | TTCTCGGACCAGCAGTGACCTT | TCAGTCCAGAGGTTTCCTCCAG |
| QPRT | GTGAAGGATAACCATGTGGTGGC | CTGCTGCATTCCACTTCCACCT |
| RHBDL3 | GAACACCTGGGACTCAATGTGG | TGTCAGCCACAGACACTGCCAA |
| SLC27A3 | TGTGGTGTCCACCAGGAAGATG | AACTGACCAGCCGAGAACTTGG |
| SLC44A1 | CCTTGTTCCACGTAGCTGGCAA | GCCTTGCTCATTCTGAACAGGAC |
| TMTC4 | TATGCCAAGCCCTGTGCTCTGA | CTCGGAAGAACAGGTTACTCGC |
| TRIB3 | GCTTTGTCTTCGCTGACCGTGA | CTGAGTATCTCAGGTCCCACGT |
| WFDC2 | CACCTTCTGCTCTCTGCCCAAT | CACAGCCATTGCGGCAGCATTT |
| Gapdh | CATCACTGCCACCCAGAAGACTG | ATGCCAGTGAGCTTCCCGTTCAG |
| Abca1 | GGAGCCTTTGTGGAACTCTTCC | CGCTCTCTTCAGCCACTTTGAG |
| Mthfd2 | GCGAATGTGCTTGGACCAGTAC | TTTGACCTGCCAGCCACTACCA |
| Hmox1 | CACTCTGGAGATGACACCTGAG | GTGTTCCTCTGTCAGCATCACC |
| Pim1 | CGCGACATCAAGGACGAGAACA | CGAATCCACTCTGGAGGACTGT |
| Ptpre | CCATCGGGATGCTCAAGTTCCT | CACAATGAAGGTGCCAGTCCGA |
